# autoBOT: evolving neuro-symbolic representations for explainable low resource text classification

**DOI:** 10.1007/s10994-021-05968-x

**Published:** 2021-04-14

**Authors:** Blaž Škrlj, Matej Martinc, Nada Lavrač, Senja Pollak

**Affiliations:** 1 Jožef Stefan Institute, Jamova 39, 1000 Ljubljana, Slovenia; 2 Jožef Stefan International Postgraduate School, Jamova 39, 1000 Ljubljana, Slovenia; 3 University of Nova Gorica, Glavni trg 8, 5271 Vipava, Slovenia

**Keywords:** Representation learning, Natural language processing, AutoML, Neuro-symbolic computing

## Abstract

Learning from texts has been widely adopted throughout industry and science. While state-of-the-art neural language models have shown very promising results for text classification, they are expensive to (pre-)train, require large amounts of data and tuning of hundreds of millions or more parameters. This paper explores how automatically evolved text representations can serve as a basis for explainable, low-resource branch of models with competitive performance that are subject to automated hyperparameter tuning. We present autoBOT (automatic Bags-Of-Tokens), an autoML approach suitable for low resource learning scenarios, where both the hardware and the amount of data required for training are limited. The proposed approach consists of an evolutionary algorithm that jointly optimizes various sparse representations of a given text (including word, subword, POS tag, keyword-based, knowledge graph-based and relational features) and two types of document embeddings (non-sparse representations). The key idea of autoBOT is that, instead of evolving at the learner level, evolution is conducted at the representation level. The proposed method offers competitive classification performance on fourteen real-world classification tasks when compared against a competitive autoML approach that evolves ensemble models, as well as state-of-the-art neural language models such as BERT and RoBERTa. Moreover, the approach is explainable, as the importance of the parts of the input space is part of the final solution yielded by the proposed optimization procedure, offering potential for meta-transfer learning.

## Introduction

Contemporary machine learning approaches successfully solve many natural language processing tasks, spanning from question answering, disambiguation, duplicate detection to classification. The emerging paradigm that successfully solves these tasks are transformer-based language models, i.e. deep neural networks that are first pre-trained on large corpora and only fine-tuned for a specific task (Devlin et al. 2019; Jing and Xu 2019).

Even though such (black-box) models offer state-of-the-art performance, the models are not directly explainable (Rudin 2019). Further, specialized hardware, such as Tensor Processing Units (TPUs) or GPGPUs (General Purpose Graphical Processing Units) are needed for their training and evaluation. Neural language models (such as the transformer architectures) inherently operate with dense vector spaces (embeddings), leveraging the multiparallelism of the modern hardware (Jouppi et al. 2017). This work focuses on the other part of the model spectrum: we investigated whether different sparse representations of text could be *evolved* in a low-resource manner, offering similar performance as dense representations, especially in settings where the available data is scarce. The main contributions of this work are summarized below.

- We propose autoBOT (automatic Bags-Of-Tokens), a system capable of efficient, simultaneous learning from multiple representations of a given document set.
- The system’s hyperparameters are optimized by using an evolutionary algorithm, adopted for exploration of high-dimensional sparse vector spaces—evolution governs the representation used for learning by a collection of linear models trained with stochastic gradient descent.
- The dimension of the evolved space is estimated based on the expected sparsity of the representation.
- The performance of autoBOT can be competitive to pre-trained transformer models and other state-of-the-art learners, as demonstrated on fourteen text classification data sets, while using less computational resources and requiring zero manual hyperparameter tuning for achieving reasonable out-of-the-box performance (given enough time).
- autoBOT offers visualization of the similarity of parts of the feature space across multiple data sets. Such visualizations offer fast overview into key parts of the feature space relevant for a given data set.
- We explore three novel feature types, namely features derived from document keywords, relational features that represent pairs of tokens at a given distance and first-order features constructed based on a collection of 34,074,917 grounded relations from the ConceptNet (Speer et al. 2017) *knowledge graph*.
- The proposed system is especially suited for settings, where hardware as well as the amount of data are limited.

The remainder of this work is structured as follows. In Section 2 we discuss the related work that influenced the development of autoBOT. Section 3 presents the proposed autoBOT system for learning from evolvable text representations, including the issue of representing texts, the formulation of the autoBOT learning task, as well as the issue of its explainability. Section 4 presents the conducted experiments, and in Section 5 we discuss the obtained results. Section 6 presents the conclusions and plans for further work.

## Related work

In this section we discuss the related approaches that inspired the development of the proposed autoBOT system. We begin by discussing the notion of text representation learning (Section 2.1), followed by text classification (Section 2.2) and evolutionary computation (Section 2.3). Finally, we discuss the state-of-the-art autoML systems in Section 2.4.

### Text representation learning

Machine learning approaches that learn from text usually consist of two main steps: preprocessing the text into a suitable representation, e.g., the Bag-of-words (BoW) format, followed by subsequent learning. The main drawback of such approaches is the requirement of the user’s specification of how the text should be represented, at what granularity etc. Such semi-automated feature construction can be time-demanding and requires large amounts of development time, however, the subsequent *learning* can be very efficient (Mirończuk and Protasiewicz 2018).

Recent developments in the field of representation learning offer many insights into the importance of having a suitable representation for the given problem. Transformer-based language models, such as BERT (Devlin et al. 2019), RoBERTa (Liu et al. 2019), XLNet (Yang et al. 2019), learn multi-faceted representations of the provided input sequences, where multiple computational layers are used to distill the obtained representation into a form used for more general problem solving. Similar insights also emerged in the fields of graph (Kipf and Welling 2017) and image (Szegedy et al. 2017) representation learning. The state-of-the-art transformer language models also use subword information due to byte-pair encoded inputs (Sennrich et al. 2016), offering even better performance, albeit at the cost of explainability.

Representations learnt by deep neural network models are dense; for example, vectors of dimension $< 1000$ are used to capture relations between input tokens. On the other hand, many shared tasks, especially the ones where the number of input instances is in the order of hundreds, yield themselves to more conventional, even linear models that operate on sparse input spaces (Martinc et al. 2017). The main caveat of such approaches is the inclusion of the human factor: humans need to *carefully* fine-tune many parameters without well defined properties or predictable behavior. For example, it is not clear how the word-based features should be weighted when compared to character-based ones, how the classifier should be regularized etc.

Further, the collections of *features* are also arbitrary as there is no general theoretical background as to when to apply what type of e.g., n-grams or other features (e.g., emoji counts etc.). Hence, such systems are commonly fine-tuned for a particular domain, yet need non-negligible human effort to perform adequately well for the same task in a different domain. For example, a system can perform well when classifying sentiment, however it fails at the prediction of side effects based on the patient reports. Finally, exhaustive search of the hyperparameter space is in most cases computationally intractable.

### Text classification

We continue the discussion by considering different machine learning approaches employed for the task of text classification, how they relate to this paper and what are their potential limitations. Text classification explores how representations of a given collection of documents can be *associated* with a given target space, such as for example a collection of genres. Broadly, text classification approaches can be split into two main groups, namely symbolic and sub-symbolic classifiers. The canonical example of symbolic learners are linear classifiers such as the logistic regression or linear Support Vector Machines, which learn to classify e.g., TF-IDF encoded documents (Manning et al. 2008; Kowsari et al. 2019; Agarwal and Mittal 2014). In recent years, however, the paradigm of neural language models has also offered state-of-the-art classifiers across multiple domains (Jing and Xu 2019). Some of the currently best-performing classifiers are commonly fine-tuned language models, pre-trained on large textual corpora (Belinkov and Glass 2019). Albeit extensive pre-training is currently inaccessible to majority of researchers, fine-tuning can be conducted with adequate off-the-shelf GPUs, and is actively employed on many e.g., shared tasks, ranging from classification of social media-related texts to classification of biomedical documents (Moradi et al. 2020). Compared to discussed approaches, which derive a representation from raw text, approaches that are able to exploit background knowledge alongside raw text are also of increasing interest and serve as one of the motivations for the proposed autoBOT. Background knowledge can be considered in many forms. Ontologies and taxonomies represent formally defined, hierarchical structures with human-defined concepts and relations between them. Some canonical examples of such knowledge sources are for example the WordNet (Fellbaum 2012) and similar taxonomies. On the other hand, *knowledge graphs* are the structures that can be defined semi-automatically, and are commonly comprised of millions of subject-predicate-object triplets. Examples of freely available knowledge graphs include the ConceptNet (Speer et al. 2017) used in this work.

### Evolutionary computation and learning

We discuss in more detail the applications and the underpinnings of evolutionary computation, and more specifically *genetic algorithms*, as this metaheuristic optimization idea was also used to guide representation learning conducted by autoBOT. Genetic algorithms have been considered for both combinatorial and continuous optimization problems in the second part of the 20th century (Mitchell 1998). Inspired by (a very basic) notion of biological evolution, these optimization algorithms often gradually *evolve* a solution via the process of intermediary evaluation, crossover, mutation and selection.

More recently, genetic algorithms (GA) evidence widespread use throughout industrial and academic projects, where GAs were successfully applied to tackle otherwise analytically intractable problems (Chambers 2000). Even though genetic and other algorithms for hard optimization problems were applied to many real-life problems, their use for improving machine learning approaches has only recently become mainstream (see Stanley et al. (2019) for an exhaustive overview); neuroevolution was already considered in 1960s, however it was computationally infeasible at the time. Neuroevolution performs well for traditional benchmark tasks, such as the knapsack problems (Denysiuk et al. 2019), but also real-life robotics problems (Zimmer and Doncieux 2017). Evolution-based approaches were also successfully adopted for the task of scientific workflow discovery (Pilat et al. 2016), offering symbolic descriptions of data mining workflows, directly applicable in practice. Neuroevolution Stanley et al. (2019) approaches have shown promising results in the domain of computer vision, where more efficient neural networks were evolved with minimal performance trade-offs (Zoph et al. 2018).

One of the early approaches on how genetic algorithms can be adopted for the feature selection purposes was proposed in Vafaie and De Jong (1998). The authors developed a system that employs a genetic algorithm to select feature subspaces useful for a decision tree classifier. They successfully showcased the performance of their approach on an eye-detection problem. The proposed autoBOT builds on a similar idea, i.e. that feature subspaces can be evolved prior to learning, however, extends the idea to multiple different instance (documents instead of images) representations, from symbolic to non-symbolic. Further, autoBOT also explores novel representation types such as e.g., knowledge-graph based features, capable of exploiting the knowledge beyond the textual training data considered.

More recent works explore how task scheduling can be tackled by employing a combination of evolution and learning (Dorronsoro and Pinel 2017). Similarly convincing results were also recently demonstrated for the task of material discovery (Jennings et al. 2019), where machine learning algorithms were used to guide the evolution, offering up to 50x speedup compared to naïve exhaustive search.

### Advancements in autoML systems

Automatic learning of machine learning pipelines has been thoroughly explored for tabular data in tools such as AutoWEKA (Thornton et al. 2013) and auto-sklearn (Feurer et al. 2019). The key idea is that parts of the learning procedure are *modularized* and automatically explored. For example, AutoWEKA and auto-sklearn employ Bayesian optimization (Snoek et al. 2012) for scalable and efficient exploration of such hyperparameter spaces. These approaches assume a tabular input, and consequently explore both the preprocessing, as well as heterogeneous ensemble construction methods that yield the best performing configuration. Another example of automated (tree-based) learning is conducted within TPOT (Olson et al. 2019), a tool for automatic construction of scikit-learn workflows specializing in tree-based learners. The main advantage of TPOT is *simplicity*—competitive results on tabular data sets can be obtained by merely running the default optimization setting for a dedicated amount of time. Development of approaches for automatic learning renders possible fast *prototyping*—instead of spending days in deciding to what extent the current data is suitable for learning—autoML systems offer quick and effortless answers to such questions, greatly speeding up the machine learning development and deployment process.

Another prominent example of the machine learning algorithm design are the automatically constructed deep neural architectures, for example, used for solving image recognition tasks (He et al. 2018). In this field of *neuroevolution* (Stanley et al. 2019) , genetic algorithms and their variations are commonly used, and were recently shown to perform better than many alternative optimization approaches. Even though evolved neural networks were shown to perform well for image data, and the majority of the remaining autoML systems focus on tabular data, we believe that research on how automatic machine learning can aid the development of algorithms that learn from texts is still scarce and worth exploring. The idea of autoML was adapted also to text domains (Madrid 2019). Similarly, Google also offers proprietary cloud-based solutions that address also the domain of natural language^1^. Learning from texts automatically is an interesting research question, especially if the hardware is not specialized for learning, and the data are scarce.

Apart from the machine learning-based approaches, explored by the evolutionary computation community, the machine learning papers that exploit evolution (or similar optimization) were developed in parallel to the aforementioned studies. For example, the implications of using evolutionary computation for the meta learning purposes on tabular data was also explored (Reif et al. 2012). They explored the performance of SVMs and random forest-based classifiers on over 100 data sets from the UCI  (Dua and Graff 2017). The authors have shown that a standard genetic algorithm already offers performance improvements. Note that the methods such as the auto-sklearn (Feurer et al. 2019), TPOT  (Olson et al. 2019) and AutoWEKA  (Kotthoff et al. 2017) also show consistent improvements of using stochastic optimization on tabular data. Further, autoML frameworks such as GAMA  (Gijsbers and Vanschoren 2019), hyperopt-sklearn  (Komer et al. 2014), ML-Plan  (Mohr et al. 2018) and OBOE  (Yang et al. 2019) all offer an optimization layer on top of an existing e.g., learning pipeline which requires hyperparameter tuning. The proposed autoBOT, albeit being conceptually similar to the work of  (Dua and Graff 2017) at the optimization level, explores how the evolution can be conducted at the representation level, which is a rather novel endeavour. Further, evolution on unstructured data such as texts is also a novelty compared to e.g., optimization for tabular classifiers.

### The rationale behind autoBOT

This work presents autoBOT, an approach for scalable, low-resource text classification that requires as little human input as possible, but nevertheless offers a decent classification performance. To our knowledge, similar approaches were explored mostly for tabular data, where the representation is already given, or for evolution of neural network architectures, where the models many times require custom hardware and are not (at all) explainable. We believe that evolution—when operating with less structured inputs such as texts—should simultaneously consider both the suitable representation and the subsequent learning, which was to our knowledge not yet explored at the scale done in this work. Further, the optimized feature space is inherently sparse, requiring an end-to-end implementation that operates with sparse matrix-algebraic operations (including learning), otherwise resulting in high dimensional dense vector spaces that require lots of computational resources. For example, considering a dense matrix of a hundred thousand features is computationally infeasible, unless sparse representation is considered.

## Learning from evolving text representations with autoBOT

**Fig. 1:**
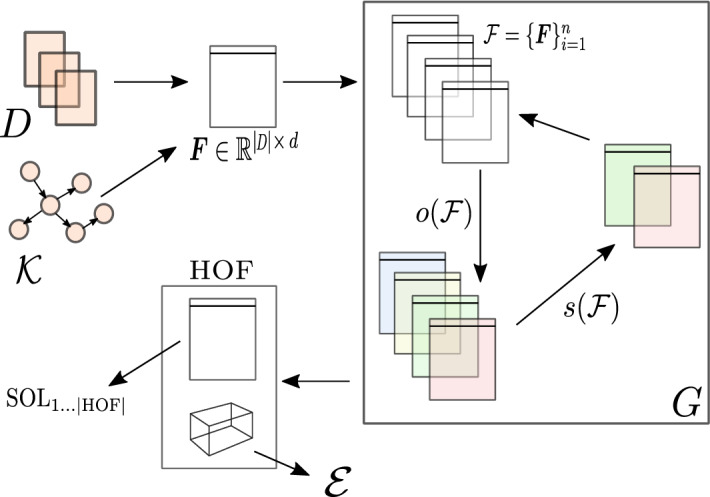
Schematic overview of autoBOT. The input is a collection of documents *D* alongside a knowledge graph ${\mathcal {K}}$. The feature space $\varvec{F}$ is constructed based on the information from both sources. Next, *G* generations of representation evolution are conducted. Here, the $o({\mathcal {F}})$ represents the application of different operators to solution vectors representing weights of feature subspaces (e.g., word, character etc.), followed by selection, $s({\mathcal {F}})$, where the next generation of solutions is chosen. Once the optimization finishes, the best solutions (HOF - Hall Of Fame) are used for the final set of predictions. The SOL_1_…_ιHOFι_ denotes the individual solutions, used for construction of final classifiers, and* ε* represents the set of explanations – feature-value associations. As the solutions encode both the weights at the feature subspace level, as well as weights of individual features, autoBOT offers two distinct views of feature importances

In this section, we present the proposed autoBOT approach. First, we discuss the representations of text considered, followed by the overall formulation of the approach. A schematic overview of autoBOT is shown in Figure 1.

Here, the training set of documents is first represented at different granularities ($\varvec{F}$); Sparse bag-of-words type of vectors on the level of characters, words, part-of-speech (POS) tags as well as keywords and relations spanning multiple tokens, to dense document embeddings and knowledge graph-based features (${\mathcal {K}}$). This is followed by the process of representation evolution (*G* field). The obtained initial set of representations is considered as the base for evolutionary optimization. Here, weights (individuals), multiplied with the feature values corresponding to the parts of this space are evolved so that a given performance score is maximized. The final set of solutions is used to obtain a set of individual classifiers, each trained on a different part of the space. However, for obtaining final predictions, a majority vote scheme is considered. Hence, evolution effectively emits an ensemble of classifiers. More details follow below.

### Multi-level representation of text

Let $\textrm {FT}$ represent the set of all *feature types* that are considered during evolution. Let *D* denote the set of considered document instances. Examples of feature types include single word features, their n-grams, character n-grams etc. Assuming *f* represents a given feature type. Let $d_f$ denote the number of features of this type. The number of all features is defined as $d = \sum _f d_f.$ Hence, the final *d*-dimensional document space consists of concatenated $\varvec{F}_f \in {\mathbb {R}}^{|D| \times d_f}$-dimensional matrices, i.e.

$$\begin{aligned} \varvec{F} = \Big |\Big |_i \varvec{F_i}, \end{aligned}$$

where *i* denotes the *i*-th feature type, and $\big |\big |$ denotes concatenation along the separate columns. The matrix is next normalized (L2, row-wise), as is common practice in text mining. Types of features considered by autoBOT are summarized in Table 1.

**Table 1:** Different feature types considered by autoBOT

Feature generator type	Description	Data type	Feature type	Sparse
Word n-grams	words	raw text	symbolic	yes
Character n-grams	tuples of sequential characters	raw text	symbolic	yes
Keyword features	one or multi-term keyphrases	graph-based token paths	symbolic	yes
Relational features	globally close characters	distance relation	symbolic	yes
POS n-grams	part-of-speech tags	grammatical	symbolic	yes
Knowledge graph features	grounded relations	semantic	symbolic	yes
Document embeddings	document embeddings (distributed memory - DM)	embedding	sub-symbolic	no
Document embeddings	document embeddings (distributed bag of words - DBOW)	embedding	sub-symbolic	no

The considered features, apart from the relational ones and document embeddings, are subject to TF-IDF weighting, i.e.,

1 $$\begin{aligned} \text {TF-IDF}(t, m) = \sum _{j \in m}\mathbb {1}[j = t] \cdot \log { \bigg ( \frac{|D|}{\sum _{k \in D}\mathbb {1}[t \in k] + 1} \bigg )}, \end{aligned}$$

where *t* is a token of interest and *m* the document of interest. The *D* is the set of all documents. While word and character n-grams, POS tags as well as document embeddings^2^ are commonly used, the relational, knowledge graph-based and keyword-based features are a novelty of autoBOT discussed below.

**Relational features**. One of the key novelties introduced in this paper is the relational feature construction method, summarized as follows. Consider two tokens, $t_1$ and $t_2$. autoBOT already considers n-grams of length 2, which would account for patterns of the form ($t_1$,$t_2$). However, longer-range relations between tokens are not captured this way. As part of autoBOT, we implemented an efficient *relation extractor*, capable of producing symbolic features described by the following (*i*-th) first-order rule: ${\mathcal {R}}_i := \text {presentAtDistance}(t_1,t_2,{{\overline{\delta }}}(t_1,t_2))$, where ${{\overline{\delta }}}$ represents the average distance between a given token pair across the *training documents*. Thus, the features represent pairs of tokens, characterized by binary feature values, derived from the top $d_{t=\text {relational}}$ distances (number of considered features) between token pairs. An example is given next.
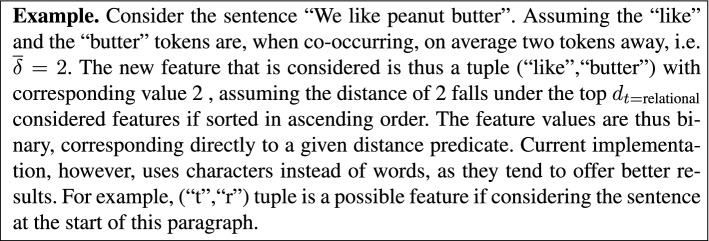


**Keyword-based features**.

The second type of features introduced in this work are the features based on *keywords*. Given a document, *keywords* represent a subset of tokens that are representative of the document. There exist many approaches for keyword detection. For example, statistical methods, such as KP-MINER (El-Beltagy and Rafea 2009), RAKE (Rose et al. 2010) and YAKE (Campos et al. 2018), use statistical characteristics of texts to capture keywords. On the other hand, graph-based methods, such as TextRank (Mihalcea et al. 2004), Single Rank (Wan and Xiao 2008), TopicRank (Bougouin et al. 2013), Topical PageRank (Sterckx et al. 2015) and RaKUn (Škrlj et al. 2019) build graphs to rank words based on their position in the graph. The latter is also the method adopted as a part of autoBOT for the feature construction process, which proceeds in the following steps:

1. **Keyword detection**. First, for each class, the set of documents from the training corpus corresponding to this class are gathered. Next, keywords are detected by using the RaKUn algorithm for each set of documents separately. In this way, a set of keywords is obtained for each target class.
2. **Vectorization**. The set of unique keywords is next obtained, and serves as the basis for novel features that are obtained as follows. For each document in the training corpus, only the keywords from the subset of all keywords corresponding to the class with which the document is annotated are recorded (in the order of appearance in the original document), and used as a token representation of a given document. This way, the keywords specific for a given class are used to construct novel, simpler “documents”. Finally, a TF-IDF scheme is adopted as for e.g., character or word n-grams, yielding *n* most frequent keywords as the final features 3.

The rationale behind incorporating keyword-based features is that more local information, specific to documents of a particular class is considered, potentially uncovering more subtle token sets that are relevant for the differentiation between the classes.

**Knowledge graph-based features**. A key novelty introduced as part of autoBOT is the incorporation of *knowledge-graph-based features*. Knowledge graphs are large, mostly automatically constructed relational sources of knowledge. In this work we explored how ConceptNet (Speer et al. 2017), one of the currently largest freely available multilingual knowledge graphs could be used to construct novel features of which scope extends the considered data set^4^. We propose an algorithm for *propositionalization* of *grounded relations*, discussed next.

Assuming a collection of documents *D*, the proposed propositionalization procedure identifies which relations, present in the knowledge graph, are also present in a given $k \in D$. Let ${\mathcal {K}} = (N,E)$ represent the knowledge graph used, where *N* is the set of terms and *E* the set of subject-predicate-object triplets, so that the subject and the object are two terms. We are interested in finding a collection of features $F_{\text {KG}}$ (i.e. knowledge graph-based features). We build on the late propositionalization ideas of Lavrač et al. (2020), where zero-order logical structures are effectively used as features, that are *automatically* identified. We refer to the algorithm capable of such scalable extraction of first-order features as PropFOL, summarised next. The key idea of PropFOL is related to grounding the triplets, appearing in a given knowledge graph while traversing the document space. More specifically, each document *k* is traversed, and the relations present in each document are stored. The relations considered by PropFOL are shown in Table 2.

**Table 2:** Considered relations. from ConcepNet considered by PropFOL

/r/Antonym	/r/AtLocation	/r/CapableOf
/r/Causes	/r/CausesDesire	/r/CreatedBy
/r/dbpedia/capital	/r/dbpedia/field	/r/dbpedia/genre
/r/dbpedia/genus	/r/dbpedia/influencedBy	/r/dbpedia/knownFor
/r/dbpedia/language	/r/dbpedia/leader	/r/dbpedia/occupation
/r/dbpedia/product	/r/Desires	/r/DistinctFrom
/r/Entails	/r/EtymologicallyDerivedFrom	/r/EtymologicallyRelatedTo
/r/ExternalURL	/r/FormOf	/r/HasA
/r/HasContext	/r/HasFirstSubevent	/r/HasLastSubevent
/r/HasPrerequisite	/r/HasProperty	/r/HasSubevent
/r/InstanceOf	/r/IsA	/r/LocatedNear
/r/MadeOf	/r/MannerOf	/r/NotDesires
/r/NotHasProperty	/r/NotUsedFor	/r/ObstructedBy
/r/PartOf	/r/ReceivesAction	/r/RelatedTo
/r/SimilarTo	/r/SymbolOf	/r/Synonym
/r/UsedFor	/r/MotivatedByGoal	/r/NotCapableOf
/r/DefinedAs	/r/DerivedFrom	

The PropFOL operates by memorizing the collections of grounded relations in each *k* (document). Once the document corpus is traversed, the *bags* of grounded relations are *vectorized* in TF-IDF manner. Finally, for each new document, two operations need to be conducted. First, the grounded relations need to be identified. Second, the collection of relations is vectorized by using the stored weights of the individual relations occurring based on the training data. The feature construction algorithm is given as the Algorithm 1.
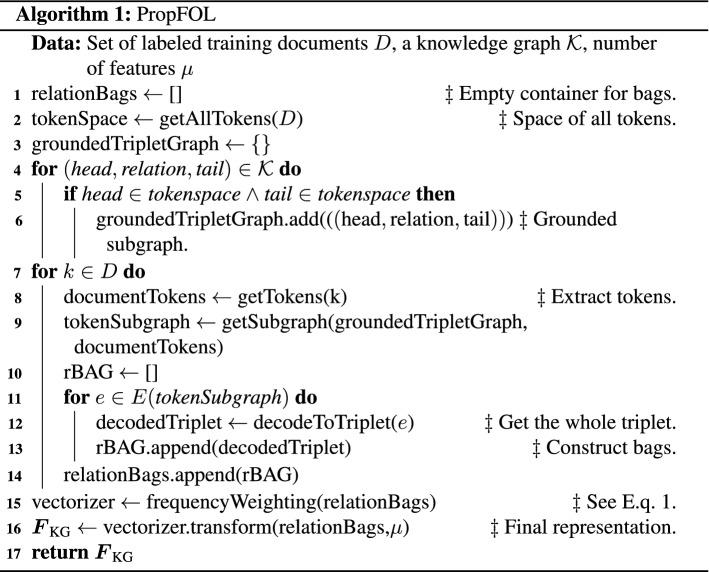
 The algorithm consists of two main steps. First, the document corpus (*D*) is traversed (line 4), whilst the relations are being recorded for each document (*k*). Once memorized (for training data, line 7), a vectorizer is constructed, which in this work conducts TF-IDF re-weighting (line 16) of first order features, and based on their overall frequency selects the top *n* such features that shall be used during evolution. Note that this simple *propositionalization* scheme is adopted due to a large knowledge graph considered in this work, as one of the key purposes of autoBOT is to maintain scalability (such graph can be processed on an off-the-shelf laptop). Note that in practice, even though millions of entities and tens of millions of possible relations are inspected, the final collection of grounded relations, particular to a considered data set, remains relatively small. In more detail, the *getAllTokens* (line 2) method maps a given document corpus *D* to a finite set of possible tokens (e.g. words). The obtained token base is retrieved for each document (*k*, line 7) via *getTokens* method. The subset of tokens corresponding to a given document is next used to extract *a subgraph* of the input knowledge graph ${\mathcal {K}}$, corresponding to a given document. This step is mandatory as the subgraph effectively corresponds to the set of triplets that are used as features. The missing component at this point are the relations, which are retrieved via the *decodeToTriplet* method (line 12). Such triplets represent potentially interesting, background knowledge (${\mathcal {K}}$)-based features. In the final part of the algorithm, triplet sets are processed as standard bags-of-items to obtain the real valued feature space suitable for learning ($\varvec{F}_{\text {KG}}$).

The following example demonstrates how the constructed features are obtained, and what are the potentially interesting relations entailed by performing such feature construction.
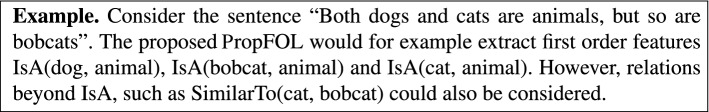


This type of feature construction is thus able to extract relations, otherwise inaccessible by conventional learners that operate solely based on e.g., word-based representations. Even though current implementation of autoBOT exploits the ConceptNet knowledge graph due to its generality, the implementation permits utilization of *any* triplet knowledge base that can be mapped to parts of texts, and as such offers many potentially interesting domain-specific applications.

### Solution specification and weight updates

The key part of every genetic algorithm is the notion of *solution* (an individual). The solution is commonly represented as a (real-valued) vector, with each element corresponding to the part of the overall solution. Let *FT* represent the set of feature types. The solution vector employed by the autoBOT is denoted with $\textrm{SOL} \in [0,1]^{|\textrm{FT}|}$ (|*FT*| is the number of feature types).

Note that the number of parameters a given solution consists of is exactly equal to the number of unique feature types (as seen in Table 1). The solution is denoted as:

$$\begin{aligned} \textrm{SOL} = \big [\underbrace{w_1,w_2, \dots , w_{|\textrm{FT}|}}_{\text {Subspace weights}}\big ]. \end{aligned}$$

Thus, the solution vector of the current implementation of autoBOT consists of 8 (hyper) parameters (for eight different feature types as seen in Table 1). Next, *solution evaluation*, the process of obtaining a numeric score from a given solution vector is discussed.

Each solution vector $\textrm{SOL}$ consists of a set of weights, applicable to particular parts of the feature space. Note that the initial feature space, as discussed in Section 3.1, consists of $d$ features. Given the weight-part of $\textrm{SOL}$, i.e. $[w_1, w_2, \dots , w_{|\textrm{FT}|}]$, we define with $I_i^\text {from}$ and $I_i^{\text {to}}$ the two column indices, which define the set of columns of the *i*-th feature type. The original feature space $\varvec{F}$ is updated as follows:

2 $$\begin{aligned} \varvec{F}^{I_i^\text {from} \text { to } I_i^\text {to}}_{s} = w_i^{s} \odot \varvec{F}^{I_i^\text {from} \text { to } I_i^\text {to}}. \end{aligned}$$

where $\odot$ refers to matrix-scalar product and *s* to a particular individual (updated feature space). Note also that the superscript in the weight vector corresponds to the considered individual. The union of the obtained subspaces represents the final *representation* used for learning.

The key idea of autoBOT is that instead of evolving on the learner level, evolution is conducted at the *representation* level. The potential drawback of such setting is that if only a single learner was used to evaluate the quality of a given solution (representation), the fitness score (that in this work equals to the mean score obtained during a five-fold cross validation on the training set) would be skewed. To overcome this issue, autoBOT—instead of a single classifier—considers a wide spectrum of linear models parameterized with different levels of elastic net regularization (trade-off between L1 and L2 norms) and losses (hinge and log loss are considered). Being trained by the stochastic gradient descent, hundreds of models can be evaluated in a matter of minutes, offering a more robust estimate of a given representation’s quality. Note that each solution is considered by hundreds of learners, and there are multiple solutions in the overall population. More formally, we denote with

3 $$\begin{aligned} {\mathcal {S}}_c(\varvec{F}) = \mathop {\mathrm {arg \, max}}\limits _{h} \big [ \textrm{SGD}(\textrm{SOL},h,\varvec{F}) \big ] \end{aligned}$$

the optimization process yielding the best performing classifier when considering feature space $\varvec{F}$, where SGD represents a single, stochastic gradient descent-trained learner parameterized via *h* (a set of hyperparameters such as the loss function and regularization). Note that SGD considers the labeled feature space during learning.

A detailed specification of the family of linear models that are considered during fitness computation are given in Section 4.2. We next discuss the final component of autoBOT that can notably impact the evolution—the initialization. Let $F_f$ represent a feature subspace (see Section 3.1 for details). The initial solution vector is specified as:

4 $$\begin{aligned} \textrm {SOL}_{\text {init}} = [{\mathcal {S}}_c(\varvec{F}_f) \cdot {\mathcal {U}}(0.95,1.05)]_{f \in FT}. \end{aligned}$$

Note the link to Equation 3: the vector consists of feature type-specific performances. The ${\mathcal {U}}(a,b)$ represents a random number between *a* and *b* drawn from the uniform distribution. This serves as noise which we add to prevent initialization of too similar individuals. As in this work the F1 score is adopted for classifier performance evaluation, its range is known (0 to 1), thus the proposed initialization offers stable initial weight setting^5^.

### Dimension estimation

Commonly, dimension of a learned representation is considered as a hyperparameter. However, many recent works in the area of representation learning indicate that high-enough dimension is a robust solution across multiple domains, albeit at the cost of additional computational complexity. The proposed autoBOT exploits two main insights and adapts them for learning from *sparse* data. The dimension estimation is parametrized via the following relation:

$$\begin{aligned} d_f = \text {round}(d_d/s), \end{aligned}$$

where $d_f$ is the final dimension, $d_d$ the *dense* dimension and *s* the estimated sparsity. The idea is that autoBOT attempts to estimate the size of the sparse vector space based on the assumption that models that operate with dense matrices require $d_d$ dimensions for successful performance, and that *s* is the expected sparsity of the space produced by autoBOT. In this work, we consider $d_d = 128$ and $s = 0.1$, the dense dimension is based on the existing literature and *s* is low enough to yield a sparse space.

### Formulation of autoBOT

Having defined the key steps for evaluation of a single solution vector $\textrm{SOL}$, we continue by discussing how such evaluation represents a part of the *evolution* process undertaken by autoBOT. The reader can observe that the genetic algorithm adopted as part of autoBOT is one of the simplest ones, introduced already in the 1990s (Davis 1991).
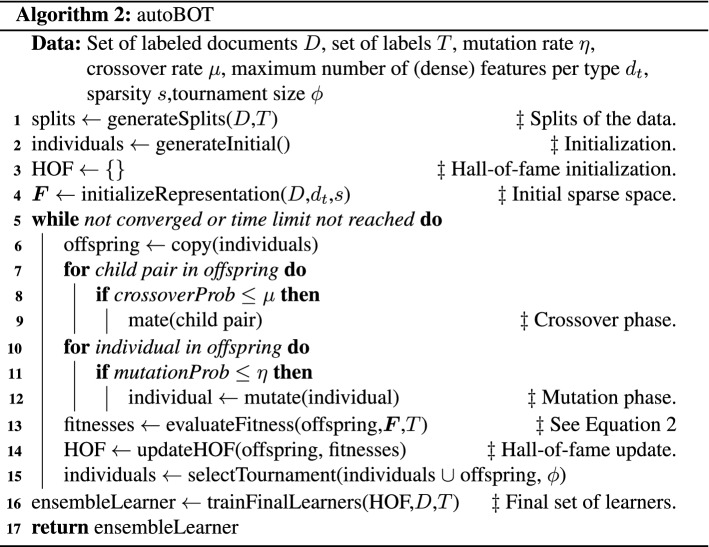


The key steps of autoBOT, summarized in Algorithm 2, are outlined below. They involve initialization (line 2), followed by offspring creation (line 6). The two steps first initialize a population of a fixed size, followed by the main while loop, where each iteration generates a novel set of individuals (solutions), and finally (line 14) evaluates them against their parents in a tournament scheme. Note that prior to being evaluated, each population undergoes the processes of crossover and mutation (lines 7 and 10), where individuals are changed either pointwise (mutation), or piecewise (crossover). Once the evolution finishes, the HOF object (hall-of-fame) is inspected, and used to construct an *ensemble* learner that performs classifications via a voting scheme. In this work, we explore only time-bound evolution. Here, after a certain time period, the evolution is stopped. The more detailed description of the methods in Algorithm 2 is as follows. The *generateSplits* method offers the functionality to generate data splits used throughout the evolution. This step ensures that consequent steps of evolutions operate on the same feature spaces and are as such comparable. The *generateInitial* method generates a collection of real-valued vectors that serve as *the initial* population as discussed in Equation 4. Next, the *initializeRepresentation* method constructs the initial feature space, considered during evolution. Note that by initializing this space prior to evolution, the space needs to be constructed only once compared to the naïve implementation where it is constructed for each individual. The *mate* and *mutate* methods correspond to standard crossover and mutation operators. The *evaluateFitness* method returns real valued performance assessment score of a given *representation*.^6^ The *updateHOF* method serves as a storage of the best-performing individuals throughout all generations, and is effectively a priority queue with a fixed size. The *selectTournament* method is responsible for comparisons of individuals and the selection of the best-performing individuals that constitute the next generation of representations. Finally, the *trainFinalLearners* method considers the best-performing representations from the hall-of-fame, and trains the final classifier via extensive grid search.

We next discuss the family of linear models considered during evolution. Note that the following optimization is conducted both during evolution (line 13) and final model training (line 16). The error term considered by stochastic gradient descent is:

$$\begin{aligned} \textrm {Err}(\varvec{w},b) = \underbrace{\frac{1}{|D|}\sum _{i=1}^{|D|} {\mathcal {L}}(\varvec{y}_i, \varvec{w}^T \varvec{x}_i + b))}_{\text {Loss term}} + \alpha \Bigg [\underbrace{ \frac{1 - \beta }{2} \sum _{i=1}^{|D|} \varvec{w}_i^2}_{\text {L2}} + \underbrace{\beta \sum _{i=1}^{|D|} |\varvec{w}_i| }_{\text {L1}} \Bigg ], \end{aligned}$$

where *y* is the target vector, $x_i$ the *i*-th instance, $\varvec{w}$ is a weight vector, ${\mathcal {L}}$ is the considered loss function, and $\alpha$ and $\beta$ are two numeric hyperparameters: $\alpha$ represents the overall weight of the regularization term, and $\beta$ the ratio between L1 and L2. The loss functions considered are the hinge and the log loss, discussed in detail for the interested reader in Friedman et al. (2001).

### Theoretical considerations and explainability

We next discuss relevant theoretical aspects of autoBOT, with the focus on computational complexity and parallelism aspects, as the no-free-lunch nature of generic evolution as employed in this work has been previously studied in other works (Wolpert and Macready 1997; English 1996). In terms of computational complexity, the following aspects impact the evolution the most:

**Feature construction**. Let $\tau$ represent the number of unique tokens in the set of documents *D*. Currently, the most computationally expensive part is the computation of keywords, where the load centrality is computed  (Škrlj et al. 2019). The worst case complexity of this step is ${\mathcal {O}}(\tau ^3)$ – the number of nodes times the number of edges in the token graph, which is in the worst case $\tau ^2$. Note, however, that such scenario is unrealistic, as real-life corpora do not entail all possible token-token sequences (Zipf’s law). The complexities of e.g., word, character, relational and embedding-based features are lower. Additionally, the features based on the knowledge graph information also contribute to the overall complexity, discussed next. Let $E({\mathcal {K}})$ denote the set of all subject-predicate-object triplets considered. The propFOL (Algorithm 1) needs to traverse the space of triplets only once (${\mathcal {O}}(|E({\mathcal {K}})|)$). Finally, both of the mentioned steps take additional |*D*| steps to read the corpus. We assume the remaining feature construction methods are less expensive.

**Fitness function evaluation**. As discussed in Section 3.2, evaluation of a single individual that encodes a particular representation is not conducted by training a single learner, but a family of linear classifiers. Let the number of models be denoted by $\omega$, the number of individuals by $\rho$, and the number of generations by |*G*| (*G* is a set of aggregated evaluations for each generation). The complexity of conducting evolution, guided by learning, is ${\mathcal {O}}(\rho \cdot \omega \cdot |G|)$.

**Initial dimensionality estimation**. The initial dimensionality is computed via a linear equation, and is ${\mathcal {O}}(1)$ w.r.t. the |*FT*| (number of feature types).

**Space complexity**. When considering space complexity, we recognize the following aspects as relevant. Let |*I*| denote the number of instances and |*FT*| the number of distinct feature types. As discussed in Section 3.1 the number of all features is denoted with $d_a$, the space required by the evolution is ${\mathcal {O}}(|I| \cdot d_a \cdot \rho )$. In practice however, the feature space is mainly *sparse*, resulting in no significant spatial bottlenecks when tens of thousands of features are considered.

The individual computational steps considered above can be summarized as the following complexity:

$$\begin{aligned} {\mathcal {O}}(\underbrace{|D| + \tau ^3 + |E({\mathcal {K}})|}_{\text {Representation construction}} + \underbrace{\rho \cdot \omega \cdot |G|}_{\text {Evolution}}). \end{aligned}$$

We next discuss how autoBOT computes solutions in parallel, offering significant speedups when multiple cores are used. There are two main options for adopting parallelism when considering simultaneously both the evolution and learning. The parallelism can be adopted either at the level of *individuals*, where each CPU core is occupied with a single individual, or at the learner level, where the grid search used to explore the space of linear classifiers is conducted in parallel. In autoBOT, we employ the second option, which we argument as follows. Adopting parallelism at the individual level implies that each worker considers a *different* representation, thus rendering sharing of the feature space amongst the learners problematic. However, this is not necessarily an issue when considering parallelism at the level of learners. Here, individuals are evaluated *sequentially*, however, the space of the learners is explored in parallel for a given solution (representation). This setting, ensuring *more memory efficient evolution*, is implemented in autoBOT. Formally, the space complexity, if performing parallelism at the individual’s level rises to ${\mathcal {O}}(c \cdot |I| \cdot d_\alpha \cdot \rho )$, which albeit differing (linearly) only by the parameter *c* (the number of concurrent processes), could result in an order of magnitude higher memory footprint (when considering autoBOT on a e.g., 32 core machine). The option with sequential processing of the individuals but parallel evaluation of learners remains of favourable complexity ${\mathcal {O}}(|I| \cdot d_\alpha \cdot \rho )$ (assuming shared memory). An important aspect of autoBOT is also explainability, which is discussed next.

As individual features constructed by autoBOT already represent interpretable patterns (e.g., word n-grams), the normalized coefficients of the top performing classifiers obtained as a part of the final solution can be inspected directly. However, in practice, this can result in manual curation of tens of thousands of features, which is not necessarily feasible, and can be time consuming. To remedy this shortcoming, autoBOT’s evolved weights, corresponding to semantically different *parts of the feature space* can be inspected *directly*. At this granularity, only up to e.g., eight different importances need to be considered, one per feature type, giving practical insights into whether the method, for example, benefits the most by considering word-level features, or it performs better when knowledge graph-based features are considered. In practice, we believe that combining both granularities can offer interesting insights into the model’s inner workings, as considering only a handful of most important low-level (e.g., n-gram) features can also be highly informative and indicative of the patterns recognized by the model as relevant.

Finally, autoBOT also offers direct insights into high-level overview of what *types of features* were the most relevant. We believe such information can serve for transfer learning purposes on the task level, which we explore as part of the qualitative evaluation.

### How successful was evolution?

Quantification of a given evolution trace, i.e. fitness values w.r.t generations has been previously considered in Beyer et al. (2002), and even earlier in Rappl (1989), where the expected value of the fitness was considered alongside the optimum in order to assess how *efficient* is the evolution, given a fixed amount of resources. To our knowledge, however, the scores were not adapted specifically for a machine learning setting, which we address in the heuristic discussed next. We remind the reader that $G = (\textrm{perf}(i))_i$ represents a tuple denoting the evolution trace – the sequence of performances. Each element of *G* is in this work a real valued number between 0 and 1. Note that the tuple is ordered, meaning that when moving from left to right, the values correspond to the initial vs. late stages of the evolution’s performance. Further, the $\textrm{perf}(i)$ corresponds to the maximum performance in each generation. Let $\max _g(G)$ denote the maximum performance observed in a given evolution trace *G*. Let ${{\,\mathrm{arg max}\,}}_g (G)$ represent the generation (i.e. evolution step) at which the maximum occurs. Finally, let |*G*| denote the total number of evolution steps. Intuitively, both the maximum performance, as well as the time required to reach such performance (in generations) need to be taken into account. We propose the following score:

$$\begin{aligned} \text {GPERF}(G) = \underbrace{\max _g (G)}_{\text {Top score}} \cdot \underbrace{\bigg (1 - \frac{{{\,\mathrm{arg max}\,}}_g (G)}{|G|} \bigg )}_{\text {How late it converged to the top score?}}. \end{aligned}$$

Intuitively, the score should be high if the overall performance is good and evolution found the best performing solution quickly. On the other hand, if all the available time was spent, no matter how good the solution, the $\textrm {GPERF}$ will be low. Note that the purpose of GPERF is to give insights into the evolution’s efficiency, which should also take into account the time to reach a certain optimum. If the reader is interested solely in performance, such comparisons are also offered. Note that $\max _g (G)$ represents the best performing solution obtained during evolution. The heuristic, once computed for evolution runs across different data sets, offers also a potential insight into how suitable are particular classification problems for an evolution-based approach – this information is potentially correlated with the problem hardness.

## Experiments

In this section we present the considered data sets, the adopted baselines with corresponding hyperparameter settings and the hardware environment used to conduct the experiments. The data sets are discussed in Section 4.1, followed by the discussion of the baselines in Section 4.2. Finally, the used hardware and software are presented in Section 4.3, followed by the evaluation in Section 4.4.

### Data sets

This section presents the data sets used for quantitative evaluation of the autoBOT’s performance. The data sets are summarized in Table 3. The selection of data sets spans from sentiment classification (*semeval* data sets), to news classification (*fox*, *bbc*), as well as personality classification (*mbti*). The data sets span various numbers of documents, from a few hundred to tens of thousands. The number of unique tokens represents the number of tokens obtained by doing document splitting directly by whitespace. Furthermore, multiclass and binary classification are considered.

**Table 3:** Summary of the considered data sets

Data set	Documents	Unique tokens	Unique labels	Task	Source
*kenyan*	462	46189	2	News source prediction	Pollak et al. (2011)
*semeval-2017-sentiment*	1156	5144	4	Sentiment prediction	Nakov et al. (2013) ^a^
*bbc*	2225	73491	4	News category prediction	Greene and Cunningham (2006)
*subjects*	1786	132996	4	Topic prediction	^b^
*fox-news*	2107	220063	7	News topic prediction	Qian and Zhai (2014)
*insults*	3946	36021	2	insult prediction	^c^
*questions*	5452	13279	6	Question types	Li and Roth (2002)
*mbti*	8675	572269	16	Personality type prediction	Myers (1962)^d^
*yelp*	10000	125446	5	Review prediction	^e^
hatespeech	10868	30555	4	Hate speech prediction	^f^
*semeval2019*	13240	53693	2	Offensive language prediction	Zampieri et al. (2019) ^g^
*sentimix*	17000	89694	3	Sentiment prediction	^h^
*articles*	19990	285167	20	Objectivity prediction	Hajj et al. (2019)
*sarcasm*	28619	58779	2	Sarcasm prediction	Misra and Arora (2019)

^a^https://bitbucket.org/ssix-project/semeval-2017-task-5-subtask-2/src/master/

^b^https://www.kaggle.com/deepak711/4-subject-data-text-classification

^c^https://www.kaggle.com/c/detecting-insults-in-social-commentary/overview

^d^https://www.kaggle.com/datasnaek/mbti-type

^e^https://www.ics.uci.edu/ vpsaini/

^f^ https://github.com/aitor-garcia-p/hate-speech-dataset

^g^ https://sites.google.com/site/offensevalsharedtask/olid

^h^https://competitions.codalab.org/competitions/20654

### Classifiers tested and hyperparameter settings

We next discuss the baseline approaches and configurations of autoBOT tested in this work. We divide baselines into the following main groups.

Manually tuned linear models. The first branch of models are linear classifiers, i.e. support vector machines (SVM) (Chang and Lin 2011) and logistic regression (LR), fine tuned across manually specified regularization ranges. The regularization of SVM and LR classifiers was in the range [0.1, 0.5, 1, 5, 10, 20, 50, 100, 500]. Each of the two learners was tested on word, character and word + character n-gram space. The feature space was normalized prior to learning.

Another autoML system. We considered TPOT, a state-of-the-art learner that adopts evolution on the level of learners (it evolves tree ensembles). We used the default settings on the word n-gram space, as this approach is not suitable for large sparse spaces.

Neural language models. Strong baselines, which operate with two orders of magnitude more parameters were also considered. More specifically, we fine-tuned BERT (base) and RoBERTa (base), two state-of-the-art language models for up to 20 epochs with early stopping, should the optimization converge faster. The hyperparameters for the two language models were left to defaults^7^.

Representation-specific baselines. One of the key experiments needed to be conducted in order to assess the performance of the evolution was that of establishing baselines that learn directly from the constructed representation, however are not subject to iterative re-weighting of the feature space. To address this problem, we implemented a cartesian product of representation-learner baselines, that offer a solid estimation of how far can e.g., a SVM get by using only the initial autoBOT representation (but no evolution). The implemented classifiers are (as named in figures): autoBOT-svm-neural (only embeddings + SVM), autoBOT-svm-neurosymbolic (full feature space + SVM), autoBOT-svm-symbolic (symbolic features + SVM), and autoBOT-lr-neural (only embeddings + LR), autoBOT-lr-symbolic (symbolic features + LR) and autoBOT-lr-neurosymbolic (full feature space + LR).

Other baselines. We implemented a stratified majority classifier^8^.

Having discussed the baseline approaches, we next discuss the considered variants of autoBOT. The main hyperparameters of evolution that we explored were the mutation rate and crossover rate. The mutation rates were varied in the range [0.3, 0.6, 0.9] and the crossover rates in the range [0.3, 0.4, 0.6, 0.9]. The tournament size was set to be integer-rounded one third of the number of individuals. Three main variants of autoBOT are reported, i.e. autoBOT-neurosymbolic, a variant where document embeddings are evolved along with the symbolic part of the feature space and autoBOT-symbolic, a variant where the document embeddings are omitted (see Table 1). Further, autoBOT-neural evolves only the two neural representations. The time for evolution was set to 8h per data set. The time was selected from a practical viewpoint; leaving an autoML running during the night instead of having an idle machine is an option that does not require any additional time allocation at the user side. The population sizes were set to 8, the same number as the number of available cores for parallel evolution (with minimal overhead). The spectrum of linear models, evaluated during fitness evaluation was specified as follows^9^. The loss functions considered were the hinge and the log loss. The learning rate of stochastic gradient descent was set to a value from the set {0.01, 0.001, 0.0001}. The elasticnet penalty was adopted, where the ratio between L1 and L2 terms was varied in the range [0, 0.1, 0.5, 0.9, 1]. Here, if this ratio was 0, the penalty would be L2, however, if the ratio was 1, L1 penalty (lasso) would be adopted.

Finally, we discuss the data set splits considered used to evaluate the aforementioned approaches. Three different splits used for evaluation are discussed next. Each data set was split to 60% training, 20% validation and 20% testing, where the validation set was used to e.g., stop the training early on convergence when considering language models, however, as autoBOT employs cross-validation for determining the best learners, training and validation were merged—a similar scenario is computationally not feasible for language models.

### Hardware and software used

The experiments were conducted using the SLING supercomputing architecture^10^. Each run was given at most 16GB of ram and 8CPU cores. autoBOT was implemented as a CPU-parallel procedure, and does not need GPU accelerators.

Additional information on the hardware used is accessible in Appendix 1. For language models benchmarks, however, specialized hardware Nvidia Tesla GPUs with 32GB of RAM (GPU) and 128GB of RAM (CPU) was used. Intentionally, we minimized the number of dependencies. Hence, Scikit-learn was used to fit linear classifiers (highly optimized) (Pedregosa et al. 2011), evolution primitives from the DEAP library (De Rainville et al. 2012) were used, and for matrix subsetting and similar linear-algebraic operations, Scipy library was adopted (Virtanen et al. 2020). The NLTK library was used for part-of-speech tagging and language parsing (Bird et al. 2009). The GENSIM library was used to obtain document embeddings (compiled versions of the algorithms) (Khosrovian et al. 2008). The language model baselines were implemented by using the PyTorch-transformers library (Wolf et al. 2020).

### Evaluation of the results

Throughout the experiments we adopted the micro F1 score for multiclass classification and F1 score for binary classification. As critical distance diagrams (Demšar 2006) are currently one of the only alternatives for simultaneous comparison of multiple classifiers across multiple data sets, we report the results by using these diagrams (for F1 and accuracy, separately) as they offer a more compact view compared to tabular results (which are reported in Appendix 2. The distance diagrams are interpreted as follows. The black lines denote the average ranks. The lower the average rank, the better the classifier. The red lines join all classifiers which are according to Friedman-Nemenyi testing part of the same significance class – there are no significant differences in their performance at ($p = 0.05$). We interpret the diagrams in alignment with the tabular results. In terms of GPERF, we visualize distributions for different data sets—such visualizations offered insights into which data sets are, given the same resources, easier or harder for the conducted evolution.

## Results

In this section we discuss the results of empirical evaluation. We first report on classification performance in Section 5.1, followed by qualitative exploration of possible transfer learning properties of autoBOT in Section 5.2, an explainability case study in Section 5.3, and case studies of evolution’s behavior in Section 5.4.

### Classification performance

**Fig. 2:**
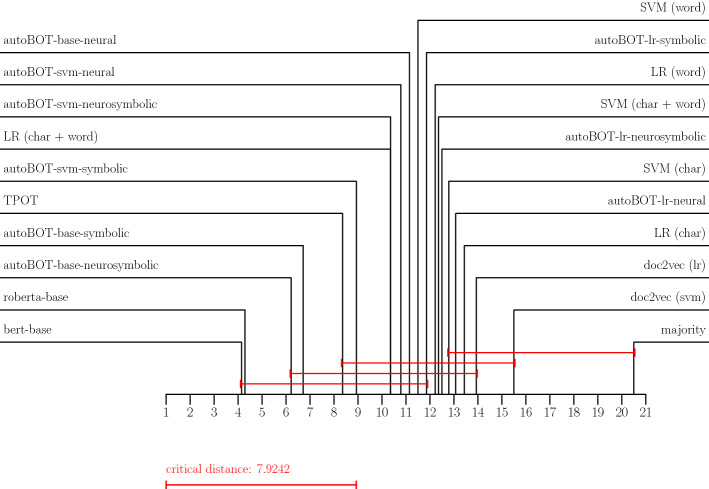
Critical distance diagrams showing average ranks based on the F1 scores

**Fig. 3:**
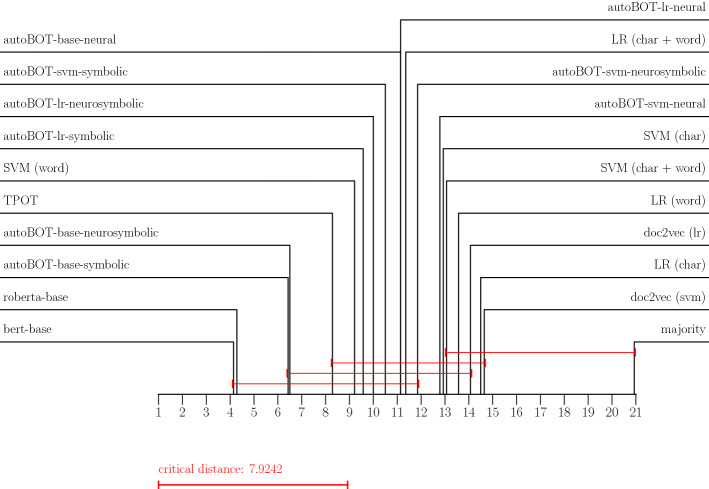
Critical distance diagrams showing average ranks based on the Accuracy scores

We summarize the F1 and accuracy-based performances in the form of critical distance diagrams, shown in Figures 2 and  3, and tabular results, shown in Tables 5 and 6 in Appendix 2. We report the results for the best performing evolution hyperparameter settings which were the mutation rate of 0.3 and the crossover rate of 0.9. It can be observed that the proposed autoBOT-neurosymbolic performs competitively to the other state-of-the-art approaches, even though it is outperformed by BERT (and to some extent by RoBERTa). Surprisingly, the symbolic-only version of autoBOT (autoBOT-symbolic) is also highly competitive. The performance is similar if compared against TPOT, and significantly higher than the weak baselines such as the majority classifier (the red lines do not join the classifiers). We also observe that RoBERTa (125M parameters) performed marginally worse than BERT (110M parameters), which we believe is due to the fact that we did not perform extensive hyperparameter search, especially exploring various regularization settings. Another interpretation of this result is that due to the large number of parameters, overfitting on the validation set occurred. Such behavior can be problematic for low resource scenarios where many classes are predicted (e.g., *mbti*). Current results indicate that language models perform sub-optimally, if multiple classes are considered (e.g., five or more), however, the results could also be due to the class imbalance, which is present in the most multiclass problems.

The overall performance can be, based on the diagrams, summarised as follows. The neural language models, as discussed, on average out-perform other approaches. The proposed autoBOT variants including either the combination of symbolic and non-symbolic features (autoBOT-neurosymbolic) and only symbolic features (autoBOT-symbolic) are ranked next, performing on average better than e.g., TPOT (autoML baseline) and other variants of linear learners trained on the constructed representation, which, however, do not consider the evolved representation. The LR (char + word) baseline performed surprisingly well, and was, out of the weaker baselines, out-performed only by the symbolic feature space of autoBOT + SVM classifier (autoBOT-svm-symbolic). The doc2vec-only representations were amongst the worst-performing ones (doc2vec (svm) and doc2vec (lr)), indicating their potential complementarity with symbolic features (as observed in e.g., autoBOT-base-neurosymbolic). Interestingly, if the two neural representations were evolved, the performance increased, however did not reach the neuro-symbolic combinations.

In terms of the performance across individual data sets, we highlight the following observations. The news-based data sets were rather easy to classify – in e.g., *bbc*, the strong learners all achieved around 99% accuracy. The data sets, where the discrepancy was larger, are for example the ones with more classes. One such example is the *mbti*, where TPOT outperformed the other learners, however was followed closely by the autoBOT-symbolic variant. On data sets such as *sarcasm*, the discrepancy between the neural language models and other types of methods was the largest. For example, BERT and RoBERTa achieved $>90\%$ accuracy, the closest autoBOT implementation was again the symbolic one which scored with 82%, which is substantially lower. Interestingly, on the data sets with a large number of instances, the proposed autoBOT came within two percentage points w.r.t. the neural language models. Finally, when considering the *hatespeech* data set, the proposed autoBOT performed on par with neural language models, albeit being completely explainable, which can be the decision factor when deploying a model on a this type of task. Overall, the clear win of neural language models is in alignment with previous work (e.g., Devlin et al. (2019)), where such models performed very well across a spectrum of multiple tasks. In terms of the interpretable methods, autoBOT was shown to offer a viable alternative a user can obtain with minimal input (and setup), and no specialized hardware (GPUs in this case).

### Towards meta transfer learning

**Fig. 4:**
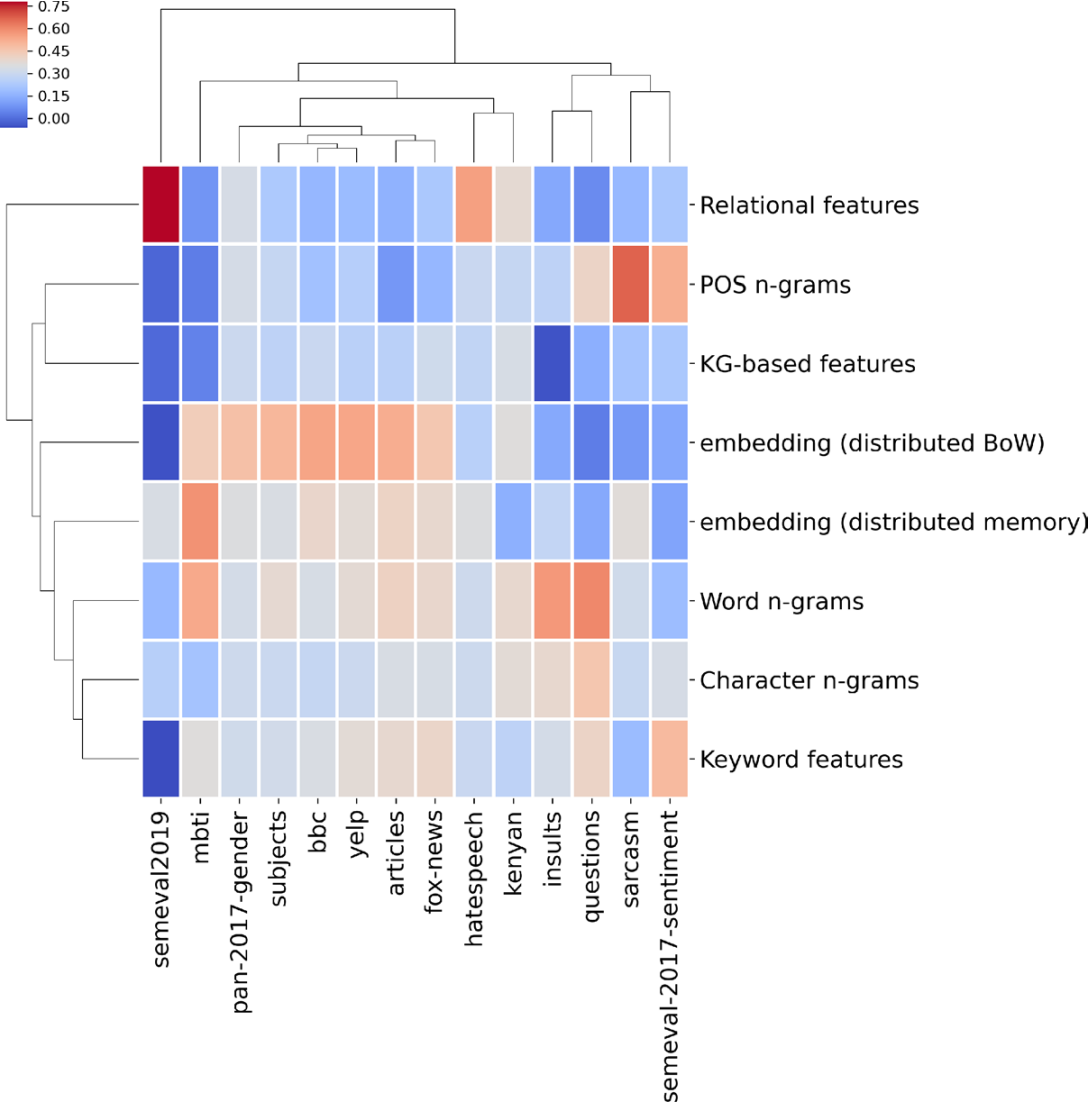
Similarity of the solution vectors across considered data sets. It can be observed that data sets related to similar tasks group together, indicating potential transfer learning possibilities at the evolution solution level. The importances were re-scaled to 0-1 range

As the proposed approach yields solution vectors that uniquely determine the importance of each type of features, we explored further whether the obtained solution vectors *share* properties across similar data sets. The clustered solution space is shown in Figure 4. The colors represent the scale of solution weights—weights that correspond to the individual feature types.

We observe that distinct clustering patterns emerge, roughly grouping the data sets based on the type of classification task. For example, the *yelp* and *bbc* data sets appear to have similar solutions, similarly the insults, questions and the sarcasm data sets. As we conducted two-way (hierarchical) clustering, insights into relations between *types* can also be observed. The POS and relational features appear to have the most in common, and similarly word-, character- and the keyword-based features. The two types of document embeddings behave similarly, and were recognized by autoBOT as such, which is an expected result that validates the purpose of such visualization. The image also offers insights into the question whether the embedding-based representations are always useful (assuming high weights correspond to relevance). For data sets such as *sarcasm* and *insults*, keyword and word-level features emerged with higher weights, however, when considering for example the *yelp* data set, the embedding-based representation appears to have had the most impact on the success of learning. Another apparent benefit of such visualization is the inspection of how relevant a given feature type is across multiple data sets. Current results indicate that POS tag-based features and the relational features appear to improve the predictive performance very selectively. For example, the POS tags appear to work well when considering the *sarcasm* data set, and relational features help, albeit moderately, when considering *semeval2019* and *hatespeech* data sets. We believe the visualizations like the proposed one are a very transparent option for *efficient exploration* of which feature types carry the most information, and could be potentially further inspected (or extended). Current results indicate that the observed clustering is related to the properties of the addressed task (e.g., embedding relevance for *bbc*, *yelp* and the *articles*)

### Explainability

One of the key features of autoBOT is its two-level transparency scheme. The first level corresponds to weights, representing parts of a given feature space, and can be used to understand what autoBOT emphasizes across data sets (Figure 4). However, autoBOT can also offer *direct importances*, based on the absolute coefficients of linear classifiers employed. An example for the *bbc* data set is given in Table 4.

**Table 4:** Top six features for different feature subspaces (*bbc* data set). The row index corresponds to considered feature types, columns are top six features. Each cell consists of the feature name and the absolute importance extracted with autoBOT. Note that even though importances can be extracted for the embedded space, they are not informative—these features are only numbered dimensions. Note that e.g., char-based features appear the same, as the difference can be in the whitespace next to a given token (part of the feature)

Index	Char features	Word features	keyword features	POS features	Relational features	KG features	Neural features v1	Neural features v2
0	film : 0.04	iaaf : 0.12	blair : 0.16	nnp nnp : 0.02	–2–e : 0.4	atlocation(committee,government) : 0.03	1951 : 1.37	3620 : 1.19
1	ilm : 0.04	mr brown : 0.07	music : 0.16	nns : 0.02	–2–n : 0.29	hascontext (fall,uk) : 0.03	3731 : 1.21	1420 : 1.18
2	mr : 0.03	drug : 0.05	brown : 0.14	cd : 0.0	e–8–l : 0.21	hascontext (mr,uk) : 0.02	1021 : 1.15	1960 : 1.09
3	fil : 0.03	mr blair : 0.05	election : 0.12	rb : 0.0	u–2–c : 0.2	relatedto (minister,british) : 0.02	4241 : 1.13	80 : 0.99
4	mr : 0.03	g8 : 0.04	athletics : 0.1	cc : 0.0	–3–l : 0.2	relatedto (secretary,government) : 0.02	1211 : 1.09	4730 : 0.98
5	mr : 0.03	mr howard : 0.04	blackpool : 0.1	ex : 0.0	a–9–o : 0.2	synonym (minister,secretary) : 0.02	4361 : 1.05	4240 : 0.97
6	fil : 0.03	rail : 0.04	party : 0.1	in : 0.0	s–7–i : 0.2	synonym (movie,film) : 0.02	4601 : 1.03	4280 : 0.95
7	mr : 0.03	wto : 0.04	straw : 0.09	nn : 0.0	–2–r : 0.18	usedfor (film,movie) : 0.02	671 : 1.02	380 : 0.94
8	mus : 0.02	big brother : 0.03	athletes : 0.08	pos : 0.0	s–6–t : 0.18	hascontext(average,uk) : 0.01	4061 : 1.0	780 : 0.91
9	mus : 0.02	hunt : 0.03	committee : 0.08	rp : 0.0	p–2–n : 0.18	hascontext(chancellor,britain) : 0.01	3711 : 0.95	2800 : 0.91

The tokens such as “blair”, “election” and similar emerged as the most relevant, which is in alignment with the task that addresses differentiation between the *topics*. Note that proper nouns (nnp – noun-noun-pronoun), either one or two in a sequence, were found to be the most relevant POS tags. The table demonstrates that even though importances can be computed for each feature separately, if the feature itself is non-symbolic, such feature importances contribute very little to the interpretation (or nothing at all). Hence, we see token or knowledge graph-level features as the most relevant when attempting to interpret what impacts the autoBOT’s decisions. Further, the proposed ConceptNet features also offer interesting insight into what predicates emerged as the most relevant. For example, *synonym(movie, film)* indicates the relevance of synonyms, however, the *hascontext(fall, uk)* offers insight into symbolic context, previously not considered in such setting.

### The Evolution’s behavior

**Fig. 5:**
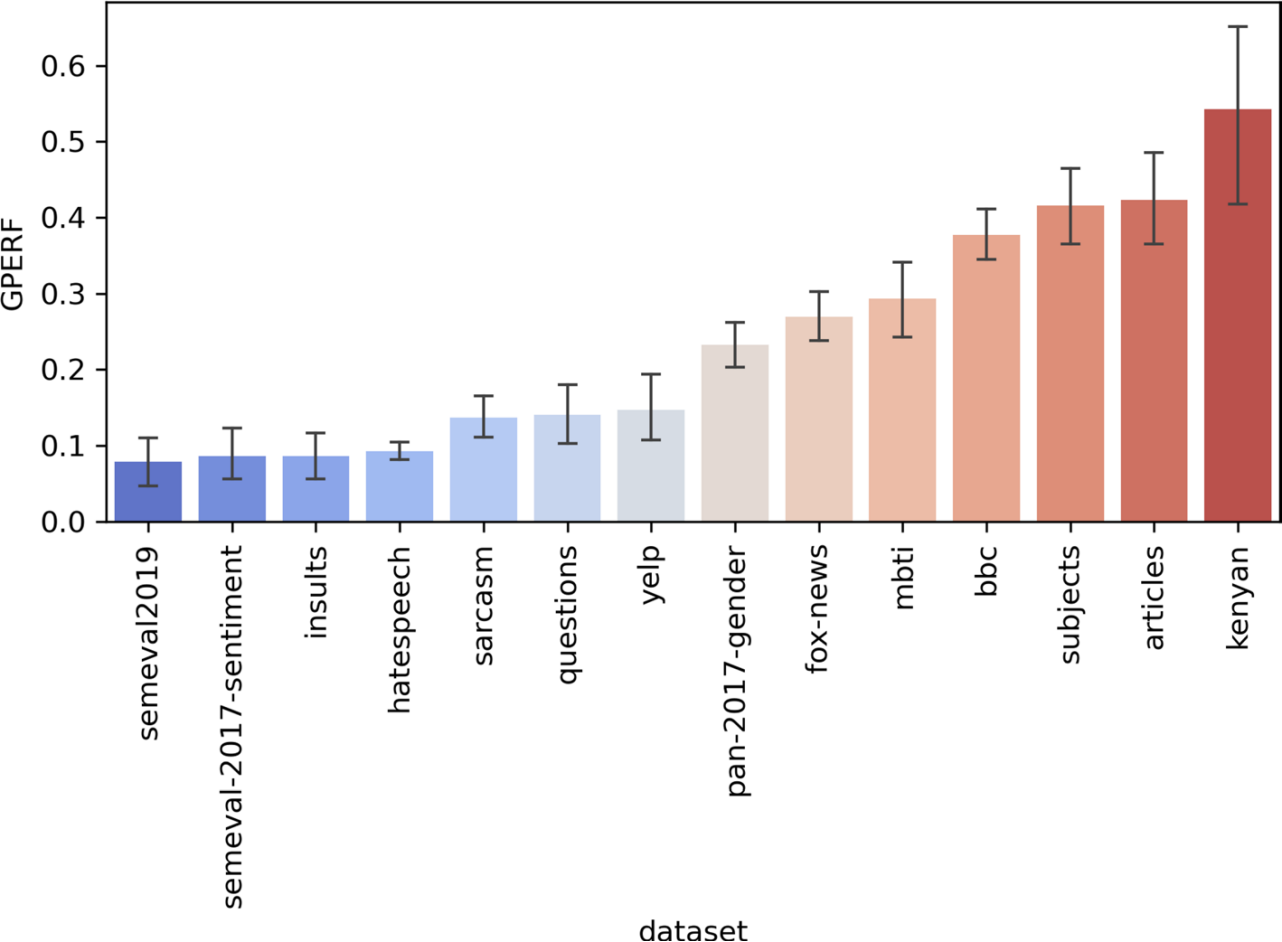
GPERF across considered data sets. The standard deviations entail different hyperparameter settings (mutation, crossover)

We next present aggregations of autoBOT’s GPERF scores when varying the evolution hyperparameters in Figure 5.

We observe the following. There exist distinct distribution differences among the data sets. For example, the *articles* and *subjects* data sets, and also *bbc* are characterized with high GPERF scores. On the other hand, *yelp*, *insults* and *semeval2019* data sets are on the lower end of the spectrum. As GPERF considers both the percentage of generations needed to convergence, as well as performance, we conjecture that the data sets with high GPERF are indeed *easier to learn*. For example, when considering *bbc*, both the F1 scores are above 95%, and also converge to the final maximum in the first couple of generations.

In contrast, we observe gradual evolution when considering e.g., the *insults* data set, and when this information is combined with the fact that F1 scores for this data set are lower than e.g., when considering *bbc*, we can conclude that this data set is harder to learn from and requires more time (generations). Another observation is that *fox*, *bbc* and *subjects* data sets are all focusing on topic prediction, where word-level semantics (and keywords) can play a dominant role. Note that comparison of multiple data sets yields different distributions even if only performances are considered—the GPERF only offers additional insight into the nature of the evolution trace that led to a certain performance. For example, the *semeval2019*’s GPERF is very low, even though its final F1 performance is around 60%. We believe GPERF (or its variants) could serve for inspecting how the evolution progresses and potentially serve as a mechanism for *automatic stopping*, however we leave such evaluation for further work. Note also, that if autoBOT would be expected to perform well on a particular collection of data sets of the same type, this type of measurement (and visualization) would offer immediate insight into its success (e.g., detection of insults, hate speech and fake news) and potentially interesting task hardness ranking.

**Fig. 6:**
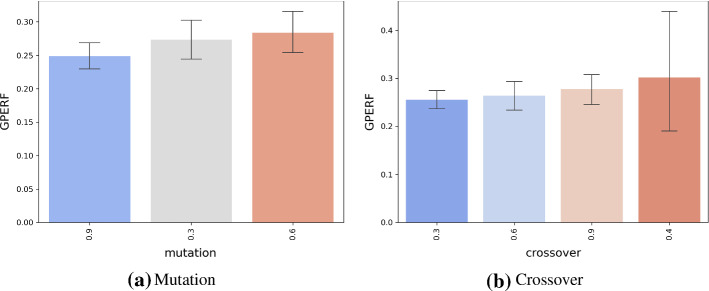
Relation between GPERF and the crossover and mutation hyperparameters of evolution. Mutation of 0.3 and crossover of 0.9 offer a good trade-off between performance and evolution convergence, and were considered as the default setting

We next discuss the behavior of the two main hyperparameters; the crossover and mutation, on the GPERF score in Figure 6. It can be observed that very high mutation rates result in, on average, lower GPERF scores (0.3 and 0.6 yield similar results). On the contrary, current results indicate that high crossover values are beneficial for the considered problem setting.

**Fig. 7:**
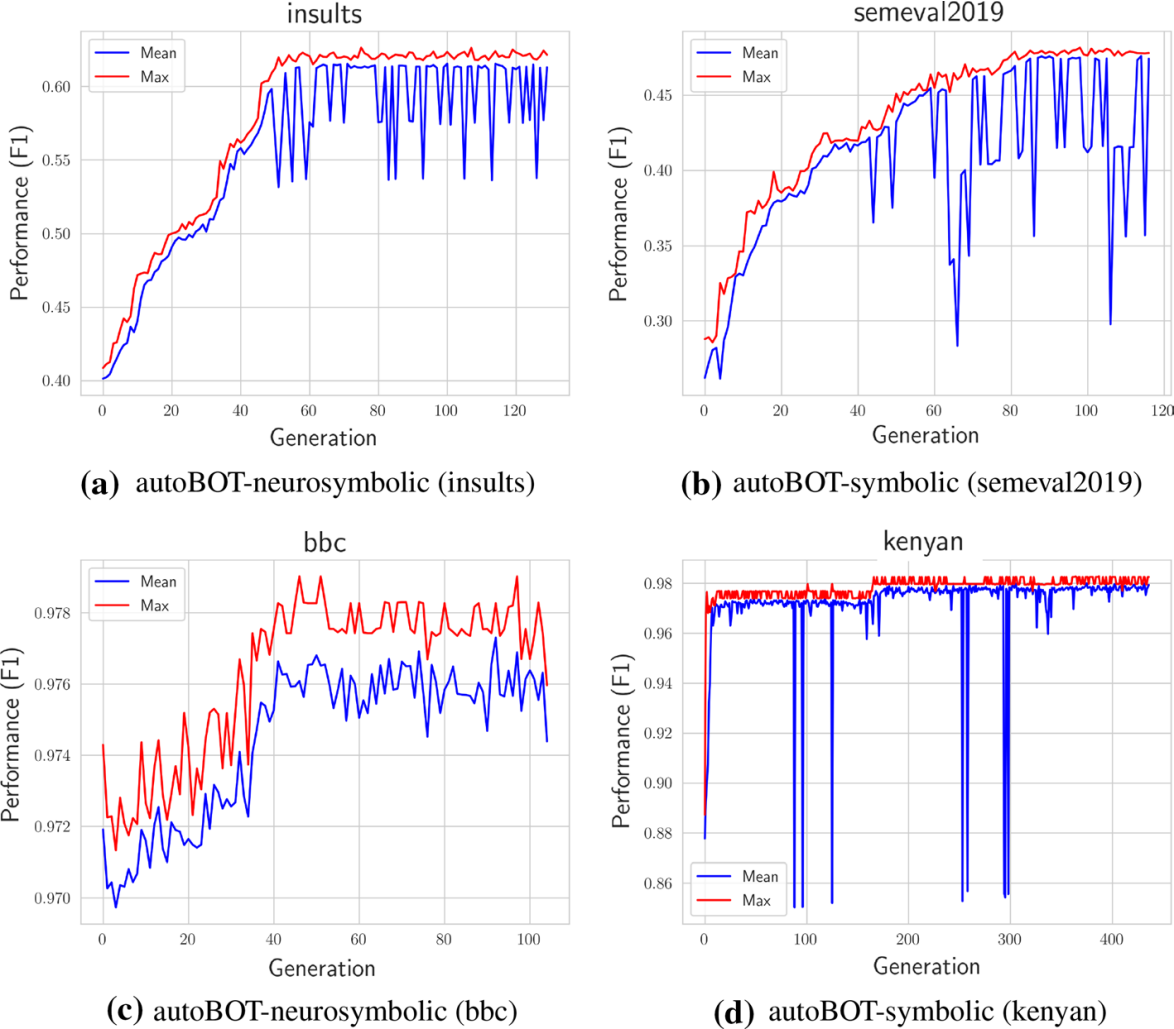
Examples of evolution traces. The blue lines represent mean and red ones maximum fitness values. It can be observed (c,b) that in some cases, the dedicated evolution time of 8 hours, was not necessarily enough to achieve convergence. On the other hand, as seen for example when considering the *kenyan* data set (d), relatively fast convergence is observed due to a relatively simple classification task. The evolution either gradually unveils a relevant representation (b), or in a few generations, as can be seen in (d)

In Figure 7 we present the interesting evolution traces we observed and discuss their implications. The figure shows four distinct evolution traces we observed when further investigating the conducted experiments. One of the key observations is that a fixed amount of time (8 hours) is not necessarily enough, and can vary highly when considering different data sets. For example, the *kenyan* data set appears relatively simple compared to e.g., the *semeval2019* data set, when gradual progress is observed, however there is no visual evidence of convergence (evolution, when considering the *kenyan* data set, converges rather quickly in the first 10% of generations). An interesting trace was observed when considering the *insults* data set, where at first larger performance increases were observed, however, when a certain point was reached, only minor improvements were present. Even though not systematically addressed, the results indicate neuro-symbolic learning is subject to *faster convergence*. Further, we acknowledge the existence of many approaches that could help with further analysis of such traces (e.g., Eiben et al. (1990)), however we consider them for further work, as the purpose of this paper was to evaluate whether autoML systems for text are feasible at all and in what scenarios.

## Discussion and conclusions

The focus of this paper is the proposed autoBOT system for automatic learning of classifiers and representations for texts. We demonstrate the system’s competitive performance on multiple data sets, when compared to strong baselines such as other autoML systems or *neural*, transformer-based language models. We additionally investigate the evolution’s behavior for selected examples, showing that instead of evolving a heterogeneous ensemble of learners, as performed by existing state-of-the-art approaches, evolution on the representation level proves to be a feasible and computationally more sensible option.

The proposed autoBOT system currently considers six symbolic and two non-symbolic document representations, however it is by no means limited to feature types considered in this work—these were selected to take multiple possible text representations into account, as well as to explore potentially interesting implications for meta transfer learning, where the solution vectors could be directly transferred across similar problems. As part of the future work, we believe incorporation of translational distance-based features could also be a promising approach. Here, a feature would be a conjunct of e.g., pairs of *presentAtDistance* predicates, which approximate the distance between the considered pair of tokens. This type of features could potentially entail more complex relations between tokens that can be otherwise hard to detect.

The proposed autoBOT approach can also be considered in analogy to the attention mechanism, used in contemporary transformer-based architectures (Devlin et al. 2019). The neural attention, during backpropagation, *prioritizes* parts of the byte pair encoded space, yielding sparse signals that are highly dependent on the context. The evolution, as implemented in this work, effectively optimizes a single vector of weights, each corresponding to a particular *collection* of features. Similarly to the attention, however, particular collections are left out (e.g., character-level features when considering semantics-rich texts). In this way, the evolution is responsible for distillation of the feature space (and not backpropagation). Finally, we believe that also the granularity of the considered space is different. While the attention mechanism emphasized e.g., individual tokens (or pairs), the autoBOT importances are related to larger feature subsets related to feature types.

Even though the proposed implementation of autoBOT is not meant for online execution, a potentially interesting research direction would be its adaptation for operation with e.g., *data streams*. Here, we see two main opportunities on how this setting could be considered. First, the existing, pre-initialized evolution weight space could be used to evolve a collection of classifiers just for a few iterations, potentially adapting to the new properties of the data, and second, as the learners are trained with stochastic gradient descent, their weights could be updated in a minibatch manner; in this scenario, the evolution iteration would not be considered after each learning update but more seldom, lifting the potentially time expensive re-training.

The proposed dimensionality estimation procedure operates based on a simple assumption that there exist useful high-dimensional feature spaces that have the same memory footprint as the commonly used low-dimensional ones (e.g., of size 128). This intuitively means that one can select the dimensions with the spatial footprint of a reasonable size, e.g., a 128 dimensional dense representation (the dimension is a hyperparameter), for which we already got an insight into its behavior on a given hardware. The estimation assumes the same dimension for all feature types, making it possible to happen that e.g., there are fewer POS-based features than the estimated dimension permits. This could be solved via some form of dynamic assignment procedure, despite the apparently low expected effect on the overall performance.

In terms of computational load, we observed the following. As the proposed autoBOT was developed with sparse representation structure in mind, its memory footprint never exceeded that of available in individual cluster jobs (16GB). As the runtime is coupled with the parameter denoting the time, current results indicate that in 8h (e.g., over-night), autoBOT is able to find good classifiers, an explanation as to what are the relevant parts of the feature space, and the features themselves that matter for the final classification. We observed that even though TPOT performs competitively, it is not able to leverage the sparseness of input matrices, resulting in potentially high memory overhead. Finally, as the neural language models were evaluated on specialized hardware, and could not be easily fine-tuned on an off-the-shelf laptop due to high working memory, disk and computation requirements, we believe this branch of models does not cover all the low-resource scenarios in which symbolic or neuro-symbolic approaches should operate well.

In terms of explainability, the proposed autoBOT offers insight into feature type and feature-level importances that are jointly learned. Potentially, a similar level of explainability can be obtained by combining explanations based on linear learners that learn based on individual features in conjunction with learners that learn on the subspaces governed by the separate feature types. The main difference between the two paradigms is that the feature-type weights are obtained by evolution, offering potentially easier incorporation of additional type-related constraints or simultaneous consideration of multiple objectives related to a given representation’s properties. The bags-of-features-based approaches can be, on the contrary, faster and are potentially an interesting future research direction in terms of weight screening prior to the main, more computationally intense evolution part. We leave a more detailed study of the explanatory power and combinations of the two paradigms for further work. Note that the evolution performs feature selection only in the scenario where the weights are exactly zero (for a given type). This type of features will be omitted entirely during classification (extreme feature discarding). In most of the experiments conducted to this end, the evolution merely re-weighted parts of the feature space, which is used in a regularization-based approach (as part of the fitness function). Even though document embeddings could be obtained with existing language models, and potentially further improve the performance, such implementation would defeat the current purpose of autoBOT, which emphasizes low resource learning. To our knowledge current state-of-the-art language models (e.g., RoBERTa) are not yet necessarily suitable for commodity hardware, even though due to increasingly more computational power, this statement might change in the future. Overall, as autoBOT was built with modular representation learning in mind, should the need arise, contextual document space could also be included as one of the considered feature types (see Section 3.1). Further, we observed that large language models struggle with problems where the amount of data is not large, and there are many classes (e.g., *mbti*). Such behaviour will be further studied, as it is not clear whether this is a general limitation.

One of the emphasis of this paper is autoBOT’s capability to operate on sparse spaces. The sparsity of the considered document representations can be the result of two different procedures. First, the classifier, evolved as part of the evolution is regularized so that it potentially prunes out parts of the feature space. One of the classifiers explored as a part of each individual is also lasso, hence the classifier-based sparseness is obtained if the classifier performs well. Further, sparseness can also be induced at the representation level by the evolution itself; here, typed parts of the feature space can be jointly neglected (weight = 0) if e.g., character-based features are non-informative.

Current autoBOT implementation considers very basic evolution principles, known for at least 30 years. This choice is intentional, aiming to demonstrate that by considering a simple tournament-based evolution with mutation and crossover, the system already offers competitive performance. An apparent direction of future work is thus to explore more advanced evolution schemes, including the exploration of Pareto optimal representations (as for example discussed by Deb and Jain (2013))—simultaneous optimization of multiple metrics could be beneficial in many real-life scenarios (Ishibuchi et al. 2008), and shall be considered in future work.

Another design choice of autoBOT was the adoption of simple, well regularized linear learners instead of more computationally intensive ones. This choice was due to the emphasis on representation evolution, which can otherwise be out-sourced to the model itself (e.g., with deeper neural network models). Furthermore, the current implementation of autoBOT offers relatively simple (drop-in replacement) exploration of more involved models, which we leave for further work.

Finally, as the main result of this work we recognize the autoBOT’s performance to offer reasonable results with *zero* human hyperparameter tuning, while at the same time offering insights into which parts of the input space, either at the level of feature types, or at the level of individual features is relevant. Even though we employed simple coefficient normalization, we believe importance assessment can already be useful for low-risk scenarios such as e.g., model debugging for news classification, however more involved normalization schemes with statistical guarantees should be adopted if systems of this type were to be used in more high-risk (e.g., biomedical) domains. The proposed implementation offers a straightforward way of obtaining relatively strong classifiers with as little human input as possible, whilst remaining interpretable.

## Data Availability

autoBOT’s repository will be available at https://github.com/SkBlaz/autoBOT.

## Appendix 1: Hardware used for neural language model training

The following is the hardware specification of the machine used for training neural language models. Note that GPUs were not used for autoBOT, as it performs as a parallel, CPU-only algorithm.

## Appendix 2: Tabular results

**Table 5:** Macro F1 performance across data sets and classifiers

dataset model	articles	bbc	fox	hatespeech	insults	kenyan	mbti	pan-2017	questions	sarcasm	semeval-2017	semeval2019	subjects	yelp
dummy-stratified	0.05 (0.0)	0.31 (0.02)	0.18 (0.01)	0.77 (0.0)	0.3 (0.02)	0.53 (0.03)	0.14 (0.01)	0.52 (0.11)	0.2 (0.01)	0.49 (0.01)	0.38 (0.01)	0.34 (0.0)	0.37 (0.03)	0.29 (0.01)
LR (word)	0.62 (0.0)	0.96 (0.0)	0.86 (0.0)	0.84 (0.0)	0.58 (0.0)	0.96 (0.0)	0.58 (0.0)	0.77 (0.24)	0.78 (0.0)	0.78 (0.0)	0.51 (0.0)	0.54 (0.02)	0.96 (0.0)	0.49 (0.0)
SVM (word)	0.63 (0.0)	0.96 (0.0)	0.86 (0.0)	0.81 (0.0)	0.63 (0.0)	0.97 (0.0)	0.65 (0.0)	0.82 (0.26)	0.82 (0.0)	0.78 (0.0)	0.45 (0.0)	0.53 (0.03)	0.95 (0.0)	0.49 (0.0)
LR (char)	0.68 (0.0)	0.95 (0.0)	0.8 (0.0)	0.83 (0.0)	0.64 (0.0)	0.95 (0.0)	0.4 (0.0)	0.8 (0.22)	0.76 (0.0)	0.77 (0.0)	0.42 (0.0)	0.54 (0.0)	0.98 (0.0)	0.48 (0.0)
SVM (char)	0.69 (0.0)	0.94 (0.0)	0.82 (0.0)	0.83 (0.0)	0.62 (0.0)	0.96 (0.0)	0.48 (0.0)	0.79 (0.24)	0.78 (0.0)	0.77 (0.0)	0.43 (0.0)	0.56 (0.04)	0.98 (0.0)	0.46 (0.0)
LR (char + word)	0.7 (0.0)	0.96 (0.0)	0.86 (0.0)	0.83 (0.0)	0.64 (0.0)	0.91 (0.0)	0.65 (0.0)	0.81 (0.26)	0.82 (0.0)	0.81 (0.0)	0.42 (0.0)	0.56 (0.0)	0.97 (0.0)	0.5 (0.0)
SVM (char + word)	0.64 (0.0)	0.95 (0.0)	0.88 (0.0)	0.81 (0.0)	0.59 (0.01)	0.97 (0.0)	0.5 (0.0)	0.75 (0.21)	0.78 (0.0)	0.8 (0.04)	0.54 (0.0)	0.56 (0.03)	0.98 (0.0)	0.43 (0.0)
bert-base	**0.84 (0.0)**	**0.99 (0.0)**	**1.0 (0.0)**	0.88 (0.0)	**0.78 (0.01)**	**1.0 (0.02)**	0.33 (0.09)	0.68 (0.16)	**0.96 (0.0)**	0.92 (0.0)	0.67 (0.01)	**0.67 (0.01)**	0.99 (0.01)	**0.58 (0.01)**
roberta-base	0.82 (0.0)	**0.99 (0.0)**	**1.0 (0.0)**	**0.89 (0.01)**	0.77 (0.01)	0.99 (0.02)	0.26 (0.07)	0.69 (0.15)	**0.96 (0.0)**	0.93 (0.0)	**0.71 (0.13)**	0.67 (0.25)	0.98 (0.0)	0.56 (0.01)
TPOT	0.64 (0.0)	0.97 (0.0)	0.93 (0.01)	0.84 (0.0)	0.62 (0.01)	0.97 (0.0)	**0.67 (0.01)**	0.82 (0.22)	0.82 (0.0)	0.8 (0.0)	0.53 (0.0)	0.40 (0.0)	0.97 (0.0)	0.53 (0.0)
doc2vec (lr)	0.65 (0.0)	0.98 (0.01)	0.77 (0.01)	0.82 (0.0)	0.39 (0.01)	0.97 (0.01)	0.54 (0.01)	0.81 (0.23)	0.47 (0.0)	0.75 (0.0)	0.34 (0.0)	0.36 (0.01)	0.95 (0.01)	0.49 (0.01)
doc2vec (svm)	0.64 (0.0)	0.97 (0.01)	0.7 (0.01)	0.82 (0.0)	0.39 (0.01)	0.95 (0.01)	0.5 (0.01)	0.79 (0.22)	0.54 (0.01)	0.76 (0.0)	0.34 (0.0)	0.37 (0.01)	0.95 (0.01)	0.47 (0.0)
autoBOT-lr-neural	0.81 (0.0)	**0.99 (0.0)**	0.85 (0.0)	0.81 (0.0)	0.28 (0.02)	0.95 (0.0)	0.57 (0.0)	0.83 (0.21)	0.53 (0.01)	0.7 (0.0)	0.45 (0.0)	0.36 (0.01)	0.98 (0.0)	0.52 (0.0)
autoBOT-svm-neural	0.81 (0.0)	0.98 (0.0)	0.84 (0.01)	0.82 (0.0)	0.49 (0.01)	0.97 (0.0)	0.59 (0.01)	0.82 (0.19)	0.56 (0.0)	0.74 (0.0)	0.47 (0.01)	0.48 (0.01)	0.98 (0.0)	0.49 (0.0)
autoBOT-lr-symbolic	0.81 (0.0)	0.98 (0.0)	0.85 (0.0)	0.81 (0.0)	0.29 (0.02)	0.96 (0.0)	0.58 (0.0)	0.83 (0.21)	0.54 (0.01)	0.7 (0.0)	0.46 (0.01)	0.37 (0.01)	0.97 (0.0)	0.52 (0.01)
autoBOT-svm-symbolic	0.81 (0.0)	0.98 (0.0)	0.84 (0.01)	0.82 (0.0)	0.48 (0.01)	0.97 (0.0)	0.59 (0.01)	0.82 (0.19)	0.56 (0.01)	0.74 (0.0)	0.47 (0.01)	0.48 (0.01)	**0.99 (0.0)**	0.49 (0.01)
autoBOT-lr-neurosymbolic	0.81 (0.0)	**0.99 (0.0)**	0.84 (0.0)	0.81 (0.0)	0.29 (0.02)	0.96 (0.0)	0.58 (0.0)	0.83 (0.21)	0.54 (0.01)	0.7 (0.0)	0.46 (0.01)	0.36 (0.01)	0.97 (0.0)	0.52 (0.0)
autoBOT-svm-neurosymbolic	0.81 (0.0)	0.98 (0.0)	0.84 (0.0)	0.82 (0.0)	0.48 (0.02)	0.97 (0.0)	0.58 (0.0)	0.82 (0.19)	0.56 (0.0)	0.74 (0.0)	0.47 (0.01)	0.47 (0.0)	0.98 (0.0)	0.49 (0.01)
autoBOT-base-neural	0.8 (0.0)	**0.99 (0.0)**	0.86 (0.01)	0.82 (0.0)	0.49 (0.08)	0.97 (0.01)	0.6 (0.01)	**0.84 (0.21)**	0.37 (0.06)	0.67 (0.02)	0.34 (0.08)	0.46 (0.06)	**0.99 (0.0)**	0.52 (0.0)
autoBOT-base-neurosymbolic	0.8 (0.01)	**0.99 (0.0)**	0.86 (0.01)	0.82 (0.01)	0.63 (0.04)	0.97 (0.01)	0.61 (0.01)	**0.84 (0.21)**	0.78 (0.02)	0.79 (0.01)	0.54 (0.03)	0.57 (0.04)	**0.99 (0.0)**	0.52 (0.01)
autoBOT-base-symbolic	0.78 (0.01)	**0.99 (0.0)**	0.88 (0.01)	0.82 (0.01)	0.66 (0.04)	0.96 (0.01)	0.63 (0.0)	0.83 (0.23)	0.79 (0.01)	0.82 (0.01)	0.56 (0.04)	0.63 (0.06)	**0.99 (0.0)**	0.48 (0.01)

**Table 6:** Accuracy performance across data sets and classifiers

dataset model	articles	bbc	fox	hatespeech	insults	kenyan	mbti	pan-2017	questions	sarcasm	semeval-2017	semeval2019	subjects	yelp
dummy-stratified	0.05 (0.0)	0.32 (0.02)	0.18 (0.01)	0.77 (0.0)	0.63 (0.01)	0.54 (0.04)	0.14 (0.01)	0.53 (0.11)	0.2 (0.01)	0.51 (0.01)	0.38 (0.01)	0.56 (0.01)	0.38 (0.03)	0.29 (0.01)
LR (word)	0.62 (0.0)	0.96 (0.0)	0.86 (0.0)	0.87 (0.0)	0.78 (0.0)	0.96 (0.0)	0.59 (0.0)	0.77 (0.24)	0.78 (0.0)	0.79 (0.0)	0.53 (0.0)	0.73 (0.01)	0.97 (0.0)	0.5 (0.0)
SVM (word)	0.64 (0.0)	0.96 (0.0)	0.86 (0.0)	0.87 (0.0)	0.84 (0.0)	0.97 (0.0)	**0.66 (0.0)**	0.82 (0.25)	0.81 (0.0)	0.8 (0.0)	0.54 (0.0)	0.73 (0.01)	0.96 (0.0)	0.51 (0.0)
LR (char)	0.68 (0.0)	0.95 (0.0)	0.8 (0.0)	0.87 (0.0)	0.83 (0.0)	0.95 (0.0)	0.43 (0.0)	0.8 (0.22)	0.76 (0.0)	0.78 (0.0)	0.53 (0.0)	0.73 (0.0)	0.98 (0.0)	0.49 (0.0)
SVM (char)	0.69 (0.0)	0.94 (0.0)	0.82 (0.0)	0.87 (0.0)	0.83 (0.0)	0.96 (0.0)	0.5 (0.0)	0.8 (0.24)	0.78 (0.0)	0.78 (0.0)	0.53 (0.0)	0.73 (0.04)	0.98 (0.0)	0.47 (0.0)
LR (char + word)	0.7 (0.0)	0.96 (0.0)	0.86 (0.0)	0.87 (0.0)	0.83 (0.0)	0.9 (0.0)	0.66 (0.0)	0.81 (0.26)	0.82 (0.0)	0.82 (0.0)	0.53 (0.0)	0.73 (0.0)	0.97 (0.0)	0.51 (0.0)
SVM (char + word)	0.64 (0.0)	0.95 (0.0)	0.88 (0.0)	0.81 (0.0)	0.79 (0.0)	0.97 (0.0)	0.5 (0.0)	0.75 (0.21)	0.78 (0.0)	0.8 (0.02)	0.54 (0.0)	0.73 (0.04)	0.98 (0.0)	0.43 (0.0)
bert-base	**0.84 (0.0)**	**0.99 (0.0)**	**1.0 (0.0)**	0.89 (0.0)	0.89 (0.0)	**1.0 (0.02)**	0.34 (0.04)	0.68 (0.15)	**0.96 (0.0)**	0.92 (0.0)	0.68 (0.01)	0.78 (0.01)	0.99 (0.01)	**0.58 (0.01)**
roberta-base	0.82 (0.0)	**0.99 (0.0)**	**1.0 (0.0)**	**0.9 (0.01)**	0.88 (0.01)	0.99 (0.02)	0.27 (0.02)	0.68 (0.12)	**0.96 (0.0)**	**0.93 (0.0)**	**0.72 (0.08)**	**0.78 (0.04)**	0.98 (0.0)	0.56 (0.01)
TPOT	0.64 (0.0)	0.97 (0.0)	0.93 (0.01)	0.87 (0.0)	0.83 (0.0)	0.97 (0.0)	0.68 (0.01)	0.83 (0.18)	0.82 (0.0)	0.8 (0.0)	0.57 (0.0)	0.7 (0.0)	0.98 (0.0)	0.53 (0.0)
doc2vec (lr)	0.66 (0.0)	0.97 (0.01)	0.77 (0.01)	0.87 (0.0)	0.76 (0.0)	0.97 (0.01)	0.53 (0.01)	0.8 (0.22)	0.47 (0.0)	0.76 (0.0)	0.51 (0.0)	0.71 (0.0)	0.95 (0.01)	0.5 (0.01)
doc2vec (svm)	0.66 (0.0)	0.97 (0.01)	0.7 (0.01)	0.87 (0.0)	0.77 (0.0)	0.95 (0.01)	0.48 (0.01)	0.78 (0.22)	0.55 (0.01)	0.77 (0.0)	0.51 (0.0)	0.72 (0.0)	0.95 (0.01)	0.49 (0.0)
autoBOT-lr-neural	0.82 (0.0)	**0.99 (0.0)**	0.85 (0.0)	0.87 (0.0)	0.77 (0.0)	0.95 (0.0)	0.62 (0.0)	0.82 (0.18)	0.53 (0.01)	0.73 (0.0)	0.55 (0.0)	0.71 (0.0)	0.98 (0.0)	0.53 (0.0)
autoBOT-svm-neural	0.81 (0.0)	0.98 (0.0)	0.84 (0.01)	0.86 (0.0)	0.78 (0.0)	0.97 (0.0)	0.6 (0.01)	0.81 (0.17)	0.56 (0.01)	0.75 (0.0)	0.53 (0.01)	0.68 (0.01)	0.98 (0.0)	0.49 (0.0)
autoBOT-lr-symbolic	0.82 (0.0)	0.98 (0.0)	0.85 (0.0)	0.87 (0.0)	0.78 (0.0)	0.96 (0.0)	0.62 (0.0)	0.82 (0.18)	0.54 (0.01)	0.72 (0.0)	0.56 (0.01)	0.71 (0.0)	0.98 (0.0)	0.53 (0.01)
autoBOT-svm-symbolic	0.81 (0.0)	0.98 (0.0)	0.84 (0.01)	0.86 (0.0)	**0.78 (0.01)**	0.97 (0.0)	0.6 (0.01)	0.81 (0.17)	0.56 (0.01)	0.75 (0.0)	0.53 (0.01)	0.69 (0.01)	**0.99 (0.0)**	0.5 (0.01)
autoBOT-lr-neurosymbolic	0.82 (0.0)	**0.99 (0.0)**	0.84 (0.0)	0.87 (0.0)	0.78 (0.0)	**0.96 (0.0)**	0.62 (0.0)	0.82 (0.18)	0.54 (0.01)	0.73 (0.0)	0.56 (0.0)	0.71 (0.0)	0.98 (0.0)	0.53 (0.0)
autoBOT-svm-neurosymbolic	0.81 (0.0)	0.98 (0.0)	0.84 (0.0)	0.86 (0.0)	0.77 (0.0)	0.97 (0.0)	0.6 (0.0)	0.82 (0.17)	0.56 (0.0)	0.75 (0.0)	0.53 (0.01)	0.68 (0.0)	0.98 (0.0)	0.49 (0.01)
autoBOT-base-neural	0.81 (0.0)	**0.99 (0.0)**	0.86 (0.01)	0.87 (0.0)	0.77 (0.01)	0.97 (0.01)	0.61 (0.01)	**0.84 (0.2)**	0.37 (0.04)	0.68 (0.0)	0.51 (0.09)	0.71 (0.0)	**0.99 (0.0)**	0.54 (0.0)
autoBOT-base-neurosymbolic	0.81 (0.0)	**0.99 (0.0)**	0.86 (0.01)	**0.87 (0.03)**	0.82 (0.02)	0.97 (0.01)	0.61 (0.01)	**0.84 (0.2)**	0.78 (0.02)	0.79 (0.01)	0.57 (0.03)	0.72 (0.05)	**0.99 (0.0)**	0.54 (0.01)
autoBOT-base-symbolic	0.79 (0.01)	**0.99 (0.0)**	0.89 (0.01)	0.86 (0.02)	0.84 (0.03)	0.96 (0.01)	0.65 (0.0)	0.83 (0.21)	0.79 (0.01)	0.82 (0.02)	0.58 (0.04)	0.77 (0.06)	**0.99 (0.0)**	0.52 (0.01)

## Footnotes

1 https://cloud.google.com/natural-language/automl/docs/beginners-guide, however this software is not open-source.

2 See Le and Mikolov (2014) for an overview of the two embedding models used. The two namings, i.e., DBOW and DM are used in the state-of-the-art implementation in Khosrovian et al. (2008).

3 The features, identified on the training set of data as relevant are also used to construct the test set’s instances.

4 September 2020 version, found at https://github.com/commonsense/conceptnet5

5 However, should a different custom score be used, it is not necessarily a sensible approach.

6 Note that each representation is evaluated by training a collection of linear classifiers in a cross-validation setting.

7 https://github.com/ThilinaRajapakse/simpletransformers

8 https://scikit-learn.org/stable/modules/generated/sklearn.dummy.DummyClassifier.html, default option

9 https://scikit-learn.org/stable/modules/generated/sklearn.linear_model.SGDClassifier.html

10 http://www.sling.si/
